# Germline and somatic mutations in patients with multiple primary melanomas: a next generation sequencing study

**DOI:** 10.1186/s12885-019-5984-7

**Published:** 2019-08-05

**Authors:** Milena Casula, Panagiotis Paliogiannis, Fabrizio Ayala, Vincenzo De Giorgi, Ignazio Stanganelli, Mario Mandalà, Maria Colombino, Antonella Manca, Maria Cristina Sini, Corrado Caracò, Paolo Antonio Ascierto, Rosanna Rita Satta, Melanoma Unit of Sassari (MUS), Maria Filomena Dedola, Salvatore Denti, Maria Antonietta Fedeli, Maria Antonietta Montesu, Stefano Profili, Tiziana Scotto, Germana Sini, Francesco Tanda, Amelia Lissia, Antonio Cossu, Giuseppe Palmieri, for the Italian Melanoma Intergroup (IMI), Paola Ghiorzo, Paola Queirolo, Pietro Quaglino, Gerardo Botti, Vanna Chiarion Sileni, Anna Maria Di Giacomo

**Affiliations:** 1 Unit of Cancer Genetics, Institute of Biomolecular Chemistry (ICB), National Research Council (CNR), Traversa La Crucca 3, Baldinca Li Punti, 07100 Sassari, Italy; 2 Department of Medical, Surgical, and Experimental Sciences, University of Sassari, Sassari, Italy; 3 National Tumor Institute “Fondazione G. Pascale”, Napoli, Italy; 4 Department of Surgery and Translational Medicine, University of Florence, Florence, Italy; 5 Department of Dermatology, University of Parma, Parma, Italy; 6 Unit of Medical Oncology, “Papa Giovanni XXIII” Hospital of Bergamo, Bergamo, Italy

**Keywords:** Skin, Cancer, Melanoma, Mutations, NGS, CDKN2A, BRAF

## Abstract

**Introduction:**

Multiple primary melanomas (MPM) occur up to 8% of patients with cutaneous malignant melanoma (CMM). They are often sporadic harbouring several somatic mutations, but also familial cases harbouring a *CDKN2A* germline mutation have been describe in Caucasian populations. The aim of this study was to investigate the incidence, the distribution patterns and the impact of known and unknown germline and somatic mutations in patients with MPM from Italy.

**Materials and methods:**

One-hundred and two MPM patients were enrolled for germline mutation analysis, and five patients with at least four MPMs were identified for somatic mutation analysis. The demographic, pathologic and clinical features were retrieved from medical records. Molecular analysis for both germline and somatic mutations was performed in genomic DNA from peripheral blood and tissue samples, respectively, through a next generation sequencing approach, using a specific multiple-gene panel constructed by the Italian Melanoma Intergroup for somatic analysis and a commercial cancer hotspot panel for somatic analysis.

**Results:**

*CDKN2A* mutations were detected in 6/16 (37.5%) and 3/86 (3.5%) MPM cases with and without family history for melanoma, respectively. Furthermore, multiple *MC1R* and, to a lesser extent, *ATM* variants have been identified. *BAP1* variants were found only in MPM patients from southern Italy. The most frequent somatic variants were the pathogenic *BRAF*^*V600E*^ and *TP53*, followed by *KIT, PIK3CA*, *KDR*, and *NRAS*. Single *APC, ERBB4, MET, JAK3* and other variants with unknown function were also detected.

**Conclusions:**

*CDNK2A* mutation is the most relevant susceptibility mutation in Italian patients with MPM, especially those with a family history for CMM. The prevalence of this mutation and other sequence variants identified in this study varies among specific sub-populations. Furthermore, some heterogeneity in driver somatic mutations between sporadic MPMs has been observed, as well as in a number of associated sequence variants the clinical impact of which needs to be further elucidated.

**Electronic supplementary material:**

The online version of this article (10.1186/s12885-019-5984-7) contains supplementary material, which is available to authorized users.

## Introduction

Cutaneous malignant melanoma (CMM) is one of the most common and continuously increasing skin cancers worldwide [1]. CMM pathogenesis is extremely complex involving genetic and environmental factors, such as specific germline and/or somatic mutations, skin color, number and type of nevi, and sun exposure [2, 3]. Most of the patients experience the occurrence of a single CMM during their life (single primary melanoma, SPM); nevertheless, multiple primary melanomas (MPMs) occur in up to 8.2% of the cases both in a synchronous or metachronous manner, and patients with five or even more MPMs have been described [4]. The expected life-time risk of an additional CMM varies between 1.3 and 8.6% in patients with a diagnosis of CMM [5].

MPMs displays the same risk factors as SPM, but environmental factors are more relevant in the pathogenesis of SPM, while genetic factors seem to be more important for MPM. Indeed, MPM has been demonstrated to involve more frequently patients with a family history for CMM than SPM [6]. The mean age at diagnosis is approximately 60 years, somewhat higher than that for SPM, and males are most frequently affected than females [7]. In most cases it is metachronous and arises in the trunk and the extremities in males and females, respectively [8]; approximately half of the subsequent lesions occur within the same anatomical region as the index melanoma [6, 7, 9, 10]. Decreasing tumor thickness in subsequent MPMs has been also reported and lower disease stage at diagnosis showed a positive prognostic significance, though outcome and survival was found not to depend on the total number of primary lesions [11, 12].

From a genetic point of view, the most impacting germline alteration in patients with MPM is the mutation of the cyclin-dependent kinase inhibitor 2A (*CDKN2A*) gene. *CDKN2A* is a recessive tumor suppressor gene that encodes two proteins: p16^INK4A^ and p14^ARF^. In physiological conditions, p16^INK4A^ inhibits protein kinase cyclin-dependent kinase 4 (CDK4)/Cyclin D1 (CCND1), which in turn affects the cell-cycle progression depending on RB (retinoblastoma susceptibility) protein, while p14^ARF^ interferes with the murine-double-minute^− 2^ (MDM2) protein, preventing the degradation of the p53 and favoring its control on cell-cycle [13]. *CDKN2A* mutations lead to uncontrolled cell-cycle progression contributing to the genesis of melanomas. The frequency of *CDKN2A* mutation is higher in MPM patients with a family history of melanoma compared to those without (35–47% vs. 3.2–15%, respectively) [14]. Furthermore, it has been shown that the *microphthalmia-associated transcription factor* (MITF) E318K variant enrichment and the presence of single nucleotide polymorphisms in the *TERT, TYRP1, MTAP, TYR* and *MX2* genes are significantly associated with the occurrence of MPM [15, 16]. Other studies reported that BRCA-associated protein 1 (*BAP1*) and protection-of-telomeres-1 (*POT1*) mutations, as well as multiple *MC1R* variants are also associated with MPM and familial melanomas [17–19]. Nevertheless, genetic testing is currently recommended only for *CDKN2A* mutations in patients with high melanoma risk, including those with MPM. The necessity for genetic testing for other low penetrance genetic alterations needs to be established.

On the other hand, MPM represents an excellent model for the study of the heterogeneity rates within the molecular mechanisms of melanomagenesis, which include several molecular targets of modern drugs like those depending on the activation of *BRAF, NRAS* and *KIT* genes [13]; knowledge of the mutational status of these genes is currently essential for the selection of the appropriate therapy, especially in complex cases with numerous MPMs.

In this study, a next generation sequencing approach was used to investigate the occurrence of germline and somatic mutations in MPM patients from Italy, with the aim to investigate the incidence, the distribution patterns and the impact of known and unknown genetic alterations in melanomagenesis.

## Materials and methods

### Patients

Two-thousand one-hundred and nine patients with CMM have been followed-up between January 2009 and June 2017 at the centers of the Italian Melanoma Intergroup participating in the study. Among them, 105 (5%) patients had a MPM, and 102 of them were enrolled (three patients refused to participate) for germline mutation analysis; five patients who had more than four sporadic MPMs were also identified for somatic mutation analysis. Demographic, clinical and morphological data were retrieved from clinical and pathology records. In particular, data regarding hair and eye colour, Fitzpatrick phototype, childhood sunburns, number of nevi and melanomas, as well as family history of CMM were collected. Nevi counts were categorized as less than 20, 21 to 100, and more than 100. Familial cases have been defined as members of a family presenting with at least three melanomas in total, irrespective of the degree of relationship of the affected members (including the MPM proband) [14]. In particular, the following criteria were used for melanoma family classification: *a*) families with at least three affected members (the MPM proband and at least two relatives with melanoma; > 4 melanomas in total), or *b*) families with two affected members (the MPM proband and at least one familial melanoma case; > 3 melanomas in total). Melanomas were considered as synchronous when a second melanoma was diagnosed during the same first observation or, at the most, within one month from the first diagnosis. Patients were informed about the aims of the study and a written consent was obtained for peripheral blood sampling and for the use of their anonymous clinical data for research purposes. The study was performed in accordance with the declaration of Helsinki, and approved by the ethical committee of the National Cancer Institute of Naples.

### Molecular analysis

**Fig. 1:**
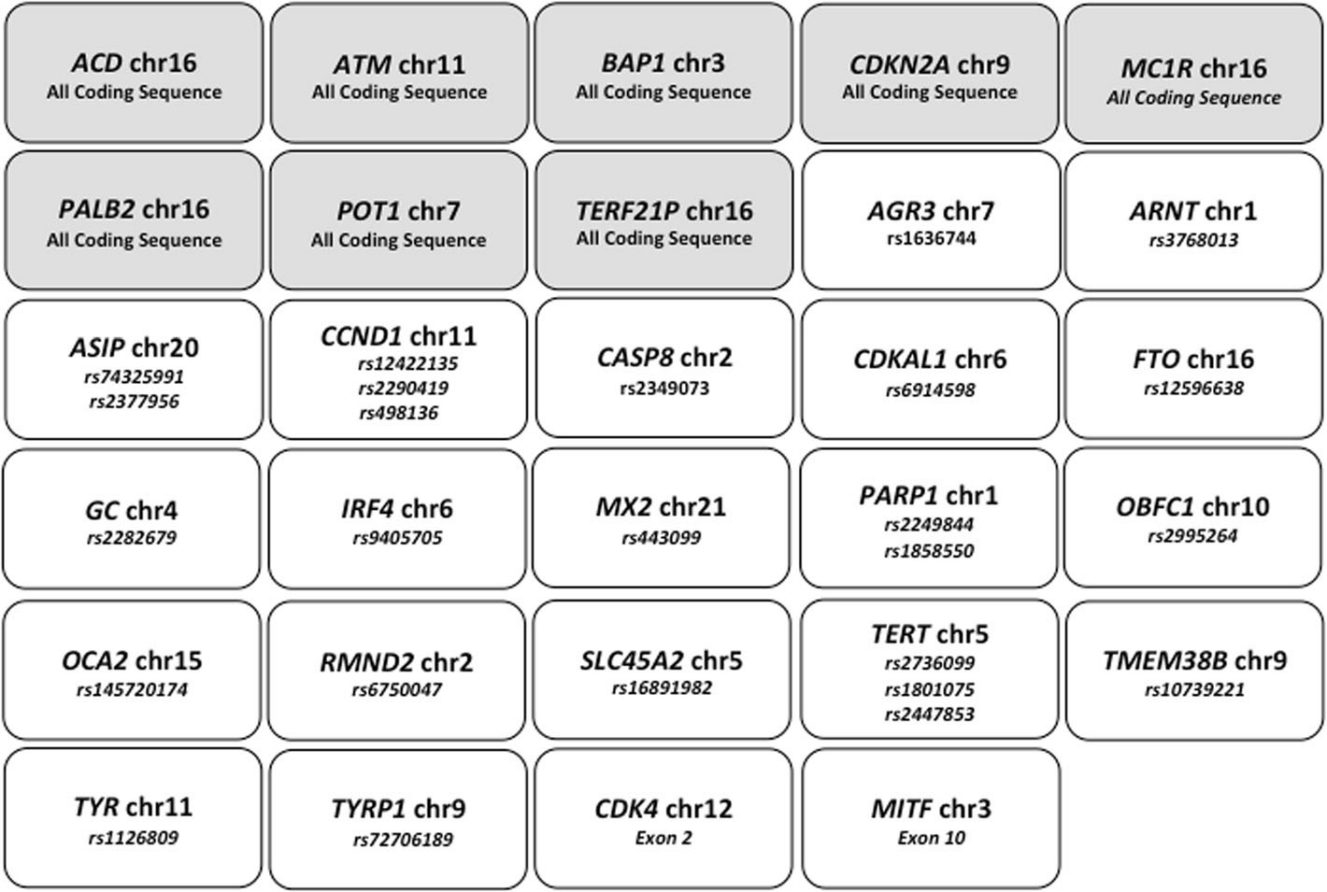
The Italian Melanoma Intergroup (IMI Germinal DNA panel) used for genetic testing. Amplicons: 190 (size range, 125–375 bp); Coverage: 99.08%; Panel size: 53.34 kb. In gray, the genes covered for the entire coding sequences

For germline mutation analysis, genomic DNA was isolated from peripheral blood samples using the QIAamp DSP DNA Blood Mini Kit (Qiagen, Hilden, Germany) according to manufacturer’s instructions. Yields of purified DNA were assessed by the Qubit dsDNA High-Sensitivity Assay Kit on the Qubit 2.0 Fluorometer (Life Thermofisher, Waltham, MA USA). The next generation sequencing (NGS) analysis was performed using the Ion Torrent PGM System with a specific multiple-gene panel constructed by the Italian Melanoma Intergroup (IMI Germinal DNA panel), arranged in two primer pools, and designed using the Ion AmpliSeq Designer to explore the mutational status of selected regions within the main 29 genes involved in melanoma susceptibility. Figure 1 summarizes the characteristics of the panel, which includes the entire coding sequences of 8 genes, the sequences of the mostly-mutated exons of 2 genes, and 25 SNPs in 19 genes (most of them in noncoding regions). Amplicon libraries were generated starting from 20 ng of genomic DNA isolated from peripheral blood, using the Ion AmpliSeq Library Kit-2.0 (Life Thermofisher), purified with Agencourt Ampure-XT Beads (Beckman Coulter, Brea, CA, USA).

For somatic mutation analysis, paraffin embedded tumor tissues of all the 28 MPMs from the five patients who had more than four sporadic MPMs were taken from the pathological archives of the institutions participating in the study. Using light microscopy, the neoplastic portion of each tissue section was selected in order to obtain tumor samples with at least 80% neoplastic cells. For mutation analysis, genomic DNA was isolated from tumor tissues, using the GeneRead DNA FFPE Kit (Qiagen, Hilden, Germany), following manufacturer’s instructions. The next generation sequencing was performed with the AmpliSeq Cancer HotSpot panel (Life Thermofisher). Each Amplicon library was prepared from a total of 10 ng template DNA and purified with AMPure beads (Beckman Coulter). The panel detects 2800 mutations in 50 genes, including all those relevant for melanomagenesis.

For both NGS-based germline and somatic analyses, purified DNA was diluted at a final concentration of 50pM, placed into the Ion Chef for emulsion PCR and Chip (316™ v2BC) loading, and sequenced on the Ion PGM using the Ion Hi-Q™ sequencing chemistry (Life Technologies). Sequencing data were processed with the Ion Torrent platform-specific pipeline software (Torrent Suite, *V5.2.1*; Life Technologies). Ion Reporter™ *V5.2* and Integrative Genome Viewer (*http://www.broadinstitute.org/igv*) were used for variant annotation and reads visualizations, respectively.

Coverage of > 100 reads and frequency of mutated alleles > 10% for gene amplicon, in order to get a total amount of > 10 mutated alleles for each candidate amplicon, were adopted for mutation selection criteria at germline level. A total of 198,395 reads was achieved for selecting 258 nucleotide variants, with an average of 769 reads per mutated gene amplicon (range, 101 to 3997). For mutation analysis at somatic level, different filtering criteria were used (after evaluating the main reports from literature on NGS-based mutation screenings): coverage of > 200 reads and frequency of mutated alleles > 3% for gene amplicon.

All sequence variants were classified as pathogenic, likely pathogenic, uncertain significance, likely benign, or benign, according to their capability to either affect the function of the gene or be plausibly linked to the disease. In particular, pathogenicity was assessed through data comparisons using the following sequence databases: the ClinVar archive of reports of relationships among medically relevant variants and phenotypes (http://www.ncbi.nlm.nih.gov/clinvar/) and the Catalogue Of Somatic Mutations In Cancer (COSMIC; https://cancer.sanger.ac.uk/cosmic).

All *CDKN2A* mutations and a large fraction of randomly-selected pathogenic mutations in the remaining genes were confirmed by Sanger sequencing of gene-specific amplicons, as previously described [20]. Briefly, polymerase chain reaction (PCR) was performed on 20 ng of genomic DNA in a Veriti 96-Well Fast Thermal Cycler (Life Technologies-ThermoFisher Scientific); all PCR-amplified products were directly sequenced using an automated fluorescence-cycle sequencer (ABI3130, Life Technologies). Sequencing analysis was conducted in duplicate and in both directions (forward and reverse) for all evaluated samples.

### Statistical analysis

Results were expressed as percentages, mean (mean ± SD) or median values (median and IQR). Variables distribution was assessed by the Shapiro-Wilk test. Statistical differences were assessed using unpaired Student’s t-test or Mann-Whitney rank sum test, as appropriate. Correlations between clinical and genetic variables were assessed by Pearson’s or Spearman’s correlation, as appropriate. Statistical analyses were performed using MedCalc for Windows, version 15.4 64 bit (MedCalc Software, Ostend, Belgium).

## Results

The Table 1 summarizes the main demographic and clinical characteristics of the patients enrolled in the study.

**Table 1:** Main clinical and epidemiological characteristic of patients with multiple primary melanomas

Characteristics	No.	%	*p* value
Total patients	102		
Gender			
Male	47	46.1	0.546
Female	55	53.9	
Median age at 1st CMM diagnosis (IQR range)			
Male	55 (40–66)	–	0.524
Female	52 (42–60)		
No. of melanomas/patients			
2	86	84.3	< 0.001
3	11	10.8	
> 3	5	4.9	
Presentation of MPMs			
Synchronous	21	20.6	< 0.001
Metachronous	81	79.4	
Incidence of 2nd melanomas			
< 1 year	37	36.3	0.098
> 1 year < 3 years	41	40.2	
> 3 years	24	23.5	
No. of total naevi			
< 20	28	27.5	< 0.001
21–100	56	54.9	
> 100	18	17.6	
Fitzpatrick phototype			
I	10	9.8	
II	41	40.2	< 0.001
III	45	44.1	
IV	6	5.9	
Sunburns in childhood			
Yes	90	88.2	< 0.001
No	12	11.8	
Family history of melanoma			
Yes	16	15.7	< 0.001
No	86	84.3	

Significance (p) has been evaluated for MPM occurrence according to each patients’ feature. *CMM* cutaneous malignant melanoma, *MPM* multiple primary melanoma, *IQR* interquartile range. Statistical significance at 0.05

Vast majority of the 102 patients enrolled had two melanomas (84.3%), and most of them (79.8%) were metachronous. A large proportion of lesions were diagnosed between the first and third year from diagnosis of the index melanoma (40.2%), mostly in patients with 21–100 nevi (54.9%). The most common phototype involved was Fitzpatrick phototype III, and 88.9% of the patients reported sunburns in childhood, while family history was reported in 15.7% of the cases.

Globally, 258 nucleotide variants were detected in the genes screened; among them, 130 (50.4%) were pathogenic in accordance with the ClinVar and COSMIC databases (see Methods). All details regarding the 258 genetic variants detected are provided in Additional file 1: Table S1. Thirty-two (31.4%) out of the 102 patients enrolled had one pathogenic mutation, 35 (34.3%) had two pathogenic mutations and nine (8.8%) had three pathogenic mutations; finally, 26 (25.5%) patients had no mutations. Table 2 summarizes the pathogenic mutations found in our study and their geographical distribution, while Table 3 illustrates their combinations in patients with more than one mutation.

**Table 2:** The pathogenic germline mutations found in our study and their geographical distribution

Genes	n° mutated cases	Central Italy	South Italy	Pathogenic variants
*CDKN2A*	8	5	3	p.G23S, p.A36T, p.A60V, p.R80*, p.R24P
*ATM*	31	10	21	p.D1853N, p.D1853V, p.L2523 M, p.L2523 fs, p.L259F, p.K2811 fs, p.F1463C, p.F858 L, p.P1054R, p.P604S,
*BAP1*	21	0	21	p.I643T
*MC1R*	57	16	41	p.R151C, p.R160W, p.D294H, p.D84E, p.Y152T*, p.V60 L, p.V92 M
*PALB2*	7	0	7	p.L1006*, p.P812S,
*TYR*	6	4	2	p.R402Q, p.P406L
Total	130	35	95

**Table 3:** Associations of the pathogenic germline variants found in our study

Genes	*CDKN2A*	*ATM*	*BAP1*	*MC1R*	*PALB2*	*TYR*	No one other
*CDKN2A*	–	3	1	7	0	0	0
*ATM*	3	–	9	16	1	0	9
*BAP1*	1	9	–	7	2	0	3
*MC1R*	7	16	7	3	3	4	16
*PALB2*	0	1	2	3	–	0	1
*TYR*	0	0	0	4	0	–	2

Among the six types of *CDKN2A* alterations detected, five were pathogenic mutations and one polymorphism (rs3731249, Table 1). The pathogenic *CDKN2A* mutations occurred in 8 (7.8%) patients; among them family history of CMM was reported in six (75%) cases, while the remaining two cases were sporadic MPMs. Considering the global cohort of 16 patients with MPM and family history of melanoma in our series, a *CDKN2A* mutation was found in the 37.5% of the cases, and thus, only in the 2.3% of the sporadic MPM cases. *CDKN2A* mutations occurred in younger patients (39.9 ± 12.9 vs 53.2 ± 15.3 years) with the age difference being statistically significant (*p* = 0.028). In addition, seven out of the eight patients (87.5%) were females, six (75%) had more than 20 nevi and all of them reported previous sunburns. The median IQR number of total family CMMs was significantly higher in patients with a *CDNK2A* mutation in comparison to those without (5, 3–6 vs. 2, 2–2 lesions, *p* > 0.001); nevertheless, the same difference was not found when the total number of personal MPMs was taken into consideration. Furthermore, two out of the eight *CDNK2A*-mutated patients and 19 out of the 94 non-*CDNK2A*-mutated were synchronous, but the difference was not statistically significant. *CDKN2A* mutations coexisted with MC1R and ATM variants in seven and three cases, respectively.

Seven pathogenic *MC1R* variants, which occurred 57 times in 53 patients, were globally found (three patients had multiple synchronous *MC1R* variants). No statistically significant differences in sex, age, phototype, childhood sunburns, family and personal number of nevi or melanomas were found in the groups of patients with and without pathogenic *MC1R* variants. Furthermore, no significant differences regarding the number of cases with family history were detected. Similar results were found for the ten *ATM* variants that occurred 31 times and the 21 *BAP1* variants observed in our cohort. The *MC1R* variants were found more frequently associated with *ATM*, *BAP1* and *CDKN2A* mutations (Table 3), while *TYR* mutations were found alone or in association with *MC1R* variants.

Among the 102 patients involved in the study, 32 were from Central Italy and 70 from the South of the country; 35 (26.9%) out of the 130 pathogenic variants found occurred in Central Italy patients and 95 (73.1%) in individuals from South Italy (Table 1). A *CDNK2A* mutation occurred in five (15.6%) cases from Central Italy and three from the South (4.3%). *TYR* mutations occurred in four (12.5%) patients form the Central and two (2.9%) patients from the South of the country. At the contrary, both *MC1R* and *ATM* variants were more common in the South than in the Central Italy. Interestingly, *BAP1* and *PALB2* pathogenic variants were detected only in Southern Italians.

The demographic, clinical and morphological data of the five patients with at least four MPMs studied for somatic mutations are summarized in Table 4. Using filtering criteria for somatic analysis (see Methods), 67 mutations were detected in the 28 MPMs examined. The most frequent mutations involved the *BRAF* and *TP53* genes. Eighteen *BRAF* mutations in 17 lesions were found in three patients; the *BRAF*^V600E^ mutation was observed in all the 17 lesions, and the rare *BRAF*^K601I^ mutation in a single case (Table 5). Wild-type *BRAF* was observed in 11 lesions; among them, nine lesions affected two patients with no BRAF mutations at all. The global frequency of lesions with *BRAF* mutations among the 28 lesions examined was, therefore, 61%. *TP53* variants were observed in 17 MPMs (again, 61%); in two lesions, two different *TP53* variants were detected, therefore the global number of *TP53* variants was 19 (Table 5). *PIK3CA* variants were found in 11 lesions (39%). Six *KDR* (21%), four *KIT* (14%), and two *NRAS* (7%) variants were also detected. Finally, single sequence variants in the *APC, ERBB4, FBXW7, JAK3, MET, SMO* and *STK11* genes were found in the cohort (Table 5; Additional file 2: Table S2).

**Table 4:** Main phenotypic and familial characteristic of patients with at least four MPMs

Case	Phototype	Hair colour	Eyes colour	Total nevi	Family member(s) with CM (No.)	Total CMs in family	Total CMs in MPM proband	Timing	Site(s) of 1st CM(s)	AJCC Stage								Germinal *CDKN2A* mutation
										1st	2nd	3rd	4th	5th	6th	7th	8th	
1	II	Light brown	Green	21–100	brother (1), sister (2)	7	4	M	Trunk	IA	IIA	IA	IIB	–	–	–	–	G23S
2	II	Light brown	Green	< 20		8	8	S	Lower limb	IB	IA*	IA*	IA	IA	IA	0	IB	wt
3	III	Dark brown	Dark brown	< 20		5	5	S	Upper limb	IB	IB	IB*	IA*	IA	–	–	–	wt
4	I	Red	Light brown	< 20	daughter (1)	6	5	S	Trunk Upper limb	IB*	IIA*	IA	IB	IA	–	–	–	wt
5	III	Dark brown	Dark brown	< 20		6	6	M	Lower limb Lower limb	IA*	IA*	IA	IB	IB	IA	–	–	wt

*CM* cutaneous melanoma, *S* synchronous, *M* metachronous, *wt* wild type; Asterisks indicate synchronous melanomas. *AJCC* American Joint Committee on Cancer

**Table 5:** The distribution of the somatic variants observed among the paired MPMs from the same patients included into the study

Patient	Sample	Sequence variants
1	M1	**CDKN2A**^**G23S**^*,* PIK3CA^I391M^*,* TP53^S99F^
	M2	**CDKN2A** ^**G23S**^ *,* **TP53** ^**E286K**^
	M3	**CDKN2A**^**G23S**^*,* TP53^P72R^
	M4	**CDKN2A**^**G23S**^*,* CDKN2A^A148T^*,* **PIK3CA**^**T1031I**^*,* TP53^P8S^
2	M1	**BRAF** ^**V600E**^ *,* **TP53** ^**R248W**^
	M2	JAK3^V722I^
	M3	**BRAF**^**V600E**^*,* ERBB4^Q264P^*,* FBXW7^T482A^*,* KDR^T875A^*,* MET^A179T^*,* SMO^L410Q^
	M4	**BRAF**^**V600E**^*,* TP53^P72A^
	M5	**BRAF** ^**V600E**^
	M6	**BRAF** ^**V600E**^
	M7	**BRAF** ^**V600E**^
	M8	**BRAF**^**V600E**^*,* TP53^S96P^
3	M1	**BRAF**^**V600E**^*,* KIT^M541L^*,* TP53^P72R^
	M2	APC^A1351T^*,* **BRAF**^**V600E**^*,* STK11^splicing^*,* TP53^H179Y^
	M3	PIK3CA^I391M^*,* TP53^P72R^*,* TP53^E286K^
	M4	**BRAF**^**V600E**^*,* PIK3CA^I391M^
	M5	**BRAF**^**V600E**^*,* KDR^G1333R^*,* PIK3CA^I391M^*,* TP53^P72R^
4	M1	**NRAS**^**G12D**^*,* PIK3CA^D1045Y^
	M2	PIK3CA^I391M^*,* PIK3CA^K468fs^*,* TP53^P72R^
	M3	**NRAS**^**G12D**^*,* PIK3CA^G903R^*,* **TP53**^**P278S**^
	M4	KDR^Q472H^*,* **PIK3CA**^**G1049S**^
	M5	KDR^Q472H^*,* PIK3CA^I391M^*,* TP53^P72R^
5	M1	**BRAF**^**V600E**^*,* KIT^M541L^*,* TP53^P72R^*,* **TP53**^R196*^
	M2	**BRAF**^**V600E**^*,* BRAF^K601I^
	M3	**BRAF**^**V600E**^*,* PIK3CA^N107H^*,* TP53^P72R^
	M4	**BRAF**^**V600E**^*,* TP53^P72R^
	M5	**BRAF**^**V600E**^*,* KDR^Q472H^*,* KIT^M541L^
	M6	**BRAF**^**V600E**^*,* KDR^Q472H^*,* KIT^M541L^

In bold, variants classified as pathogenic/likely pathogenic mutations

## Discussion

The *CDKN2A* gene is located in the 9p21 locus and represents currently the main high-risk gene predisposing to CMM, firstly assigned in familial melanoma in early nineties [21, 22]. Since then, a great amount of studies investigating the role of *CDKN2A* mutations in the genetic susceptibility of melanoma have been made. Also in our study, performed for the first time with a comprehensive panel of main genes involved in melanoma susceptibility, *CDKN2A* mutations were the most relevant disease-predisposing genetic alterations, occurring in the 37.5% of MPM patients with a family history of CMM; furthermore, 75% of the patients with a *CDKN2A* mutation had a familial MPM. This figures are similar to those reported in the scientific literature in other Caucasian populations, and in previous studies performed in Italy [6, 23]. Nevertheless, the frequency of *CDKN2A* mutations in sporadic MPMs was somewhat lower in our cohort (2.3%) than in previous studies reporting percentages ranging between 3.2 and 15% [24–26]. Finally, the global number of pathogenic *CDKN2A* mutations found in our cohort (7.8%) was similar to those reported in other studies in western countries [23, 27] but lower than figures reported in recent Italian studies prevalently including patients from North Italy [14, 26, 28–32].

This finding probably depends on differences in *CDNK2A* susceptibility patterns throughout the country. Previous studies performed in Ligurian melanoma families showed that founder *CDKN2A* mutations were prevalent in up to 40% of the cases, leading national scientific societies to recommend genetic testing in high-risk patients for familial CMM [29, 32]. Nevertheless, studies in South-Italian populations reported discrepant results. Di Lorenzo et al. screened a total of 48 familial CMM Sicilian patients for germline mutations in *CDKN2A* and *CDK4* genes; they found that none of the examined families carried mutations in exon 2 of *CDK4* and only one patient harboured a rare missense mutation in exon 2 of *CDKN2A* (2.1%) [33]. Another study was performed in Sardinia island including 24 family cases of CMM; again, only one (4.2%) *CDKN2A* mutation was detected [1]. The *CDKN2A* prevalence among Sicilians and Sardinians - which are genetically different from other European populations because of their particular geographical and historical background - rises some concerns about the effective usefulness of genetic testing in high-risk CMM patients from both islands. Moreover, recent studies performed in Central Italy institutions reported *CDKN2A* frequencies in-between those observed in the opposite poles of the country [34], depicting in some way a prevalence gradient, characterized by decreasing values from North to South Italy. Such a prevalence gradient may reflect also in MPM cases, explaining the differences between the mutation prevalence found in our cohort and that of other northern studies. Bruno et al. reported that the highest mutation rate in MPM cases was found in the northern regions of Italy, particularly in Liguria and Lombardy (35, and 24%, respectively), whereas the frequency decreased in central regions, although remaining near 10% [31]. In an older article published by our group including MPM patients from Central and South Italy, the frequency of *CDKN2A* mutations found was 13.2%, but the number of patients from South Italy was extremely low [35]. This figure is very similar to that found in the current study in patients from Central Italy (15.6%), and consistently higher from that observed in those from the South (4.3%), confirming the prevalence gradient mentioned above.

*CDKN2A* mutations in our cohort occurred in younger patients with MPM, prevalently females, reporting a high number of family lesions and childhood sunburns; these findings are widely reported in previous studies, with the exception of the high incidence rates found in females [36]. In all the cases, the mutations were associated to at least one genetic alteration in one other of the remaining genes examined, suggesting multiple interactions in determining the genetic susceptibility to melanoma. In most cases the association was with *MC1R* variants (Table 3), which in turn, have been demonstrated to be associated to a higher risk of melanoma in numerous studies [37, 38]. Some *MC1R* variants are associated with red hair colour and fair phenotype, but they have been found associated with melanoma also in South European individuals with dark/olive phenotype [39]. Ghiorzo et al. studied 49 CDKN2A-positive and 390 *CDKN2A*-negative Italian patients with CCM; *MC1R* variants were associated with increased odds of melanoma only in CDKN2A-negative patients, while first-degree family history of cutaneous melanoma increased the odds of developing melanoma in both variant-positive patients [40]. In our study, cases with both *CDNK2A* mutations and *MC1R* variants (*N* = 7) were observed in significantly younger patients with family history for CMM. Godstein et al. described a statistically significant decrease in median age at diagnosis as numbers of *MC1R* variants increased in *CDKN2A*-positive patients, but we were not able to adequately measure this feature given the small number of cases in our cohort [19]. As opposed to *CDNK2A* mutations, *MC1R* variants were more common in individuals from South Italy (difference was not statistically significant), a geographical area where *CDNK2A* mutations have been reported at lower prevalence [28, 41]. The pathophysiological role of *MC1R* remains to be better evaluated in order to determine any putative recommendation for its genetic testing.

A further interesting finding is the exclusive occurrence of *BAP1* pathogenic variants in patients from South Italy. *BAP1* is located in the 3p21 region and encodes a deubiquitylase that participates in multi-protein complexes regulating key pathways including cell cycle, differentiation and death. *BAP1* germline mutations have been associated with a syndromic disease characterized, among others, by the presence of CMM, uveal melanoma, mesothelioma, renal cell carcinoma, and other cutaneous neoplasia [36]. O’Shea et al. in a population-based study in the United Kingdom identified 22 *BAP1* variants in 1977 melanoma cases (5 variants in controls and 3 common SNPs), with a missense change (S98R) completely abolishing BAP1 activity suggestive of melanoma-predisposing *BAP1* mutation [17]. The Authors concluded that deleterious/damaging *BAP1* germline mutations in patients with CMM are rare [17]. In our study, no cases harbouring the S98R-variant were found, but only patients with I643T-variant, often associated with other mutations. The clinical significance of this finding warrants further evaluation, in order to establish the need for genetic test in populations with high prevalence of this variant. Currently, the National Comprehensive Cancer Network (NCCN) reports that BAP1 testing may be warranted in specific cases, along with testing for other melanoma-predisposing genes like *CDK4*, *MITF* and *TERT* [42]. No pathogenic germinal mutations in the latter genes were detected in our series.

Our study evidenced a very high incidence rate of *BRAF* somatic mutations (61%) and a very low prevalence of RAS mutations (7%) in the 28 sporadic MPMs evaluated. Among the 18 *BRAF* mutations encountered, 17 were V600E, which is the most common mutation in CMM, and one was K601I, a very rare pathogenic mutation according to the COSMIC database. In an older study, we analysed the *BRAF* mutational status in 112 MPM patients (96 with two, 15 with three and one with four MPMs) [9]; *BRAF* mutations were detected in 48% of the 229 primary lesions examined, which is in accordance with figures of sporadic CMM in the general population, and consistently lower with those found in our study. We reported similar results in a subsequent study among 24-paired MPMs in twelve patients [7]. The concordance in *BRAF* mutations between the index and subsequent melanomas in these studies was low, as in other literature reports [43]. The differences in the incidence of *BRAF* mutations may be due to different selection criteria (patients with familiar MPM or *CDKN2A* mutations were included), the fact that most patients enrolled had only two lesions, and differences in sequencing technology.

Nineteen *TP53* variants were found in 17 of the MPMs examined. Silencing of this gene leads to reduction of the p53 protein, contributing in boosting the aggressiveness of the tumor and its refractoriness to therapies; therefore, knowledge of its mutational status is crucial for the clinical management of CMM. Among the seven types of *TP53* variants detected, only three are classified as pathogenic in the COSMIC database. Furthermore, a pathogenic *KIT* variant was found in four MPMs, as well as several *KDR* and *PIK3CA* neutral or unknown function variants. Finally, seven very rare sequence variants were identified, distributed in 3 MPMs of two patients. Most of these variants are not included in the COSMIC database, and their functional significance is unclear.

Our study has some limitation as it is not a population-based study that includes a relatively restricted number of patients, and as a consequence, a low number of mutations detected, limiting the statistical analyses. On the other hand, it is the first study performed with wide panels of genes known to impact the pathogenesis of melanoma in MPM cases, both at a germinal and somatic level.

## Conclusions

The *CDNK2A* mutation is the most impacting germline mutation in Italian patients with MPM and a family history for melanoma, and in a relatively low percentage of patients with sporadic MPM. Nevertheless, the prevalence of this mutation is extremely low in patients with MPM from South Italy. On the other hand, multiple *MCR1* and *ATM* variants and other low penetrance mutations, like *BAP1* and *TYR* variants, have been identified with a variable prevalence among specific sub-populations. These findings suggest that genetic test for *CDNK2A* mutations in cases with family MPMs should be advised, while the clinical usefulness of genetic tests for specific lower penetrance mutations should be further investigated. In addition, a low level of heterogeneity in driver somatic mutations in patients with numerous MPMs was found. Nevertheless, their occurrence, along with that of associated somatic mutations in genes with unknown function, is unpredictable and molecular analysis in every single MPM should be carried out.

## Additional files

Additional file 1: **Table S1.** The 258 germinal variants found in our study, in detail. In bold, variants classified as pathogenic/likely pathogenic mutations. (PDF 140 kb)

Additional file 2: **Table S2.** The 73 somatic variants found in our study, in detail. In bold, variants classified as pathogenic/likely pathogenic mutations. (PDF 76 kb)

## Data Availability

The datasets used and/or analysed during the current study are available from the corresponding author on reasonable request.

## Abbreviations

AJCC: American Joint Committee on Cancer
ATM: Ataxia-Telangiectasia Mutated serine/threonine kinase
BAP1: BRCA1-associated protein-1
CDKN2A: Cyclin-dependent kinase inhibitor 2A
CMM: Cutaneous malignant melanoma
COSMIC: Catalogue for somatic mutations in cancer
DCK4: Cyclin-dependent kinase 4
IMI: Italian Melanoma Intergroup
MC1R: Melanocortin 1 receptor
MITF: Microphthalmia-associated transcription factor
MPM: Multiple primary melanoma
MTAP: S-methyl-5′-thioadenosine phosphorylase
NGS: Next-generation sequencing
PALB2: Partner and localizer of BRCA2
PCR: Polymerase chain reaction
POT1: Protection of telomeres homolog 1
SNP: Single nucleotide polymorphism
SPM: Single primary melanoma
TERT: Telomerase reverse transcriptase
TYR: Tyrosinase
TYRP: Tyrosinase-related protein
