# Supplementary material for: Germline and somatic mutations in patients with multiple primary melanomas: a next generation sequencing study
Source: BMC Cancer. 2019 Aug 5;19:772. doi: 10.1186/s12885-019-5984-7 (PMC6683413; doi:10.1186/s12885-019-5984-7)
Supplement: Supplementary file 1 — Table S1. The 258 germinal variants found in our study, in detail. In bold, variants classified as pathogenic/likely pathogenic mutations. (PDF 140 kb) [file 12885_2019_5984_MOESM1_ESM.pdf]

Case No.	Locus	Genotype	Ref	Type	Gene	Location	Exon	Coding	Protein	Variant Effect	ClinVar	COSMIC PREDICTION	Coverage	Allele 1 Type	Allele 1 Coverage	Allele 2 Type	Allele 2 Coverage	% Mutated Allele 2	Allele Ratio	p-value
1	chr11:108175462	G/A	G	SNV	ATM	exonic	37	c.5557G>A	p.Asp1853Asn	missense		Pathogenic	465	G	254	A	211	45,4	G=0.5462, A=0.4538	0.00001
1	chr16:89985940	G/A	G	SNV	MC1R	exonic	1	c.274G>A	p.Val92Met	missense	pathogenic		324	G	186	A	138	42,6	G=0.5741, A=0.4259	0.00001
1	chr16:89986154	G/A	G	SNV	MC1R	exonic	1	c.488G>A	p.Arg163Gln	missense			271	G	149	A	122	45,0	G=0.5498, A=0.4502	0.00001
3	chr11:108143456	C/G	C	SNV	ATM	exonic	22	c.3161C>G	p.Pro1054Arg	missense		Pathogenic	269	C	141	G	128	47,6	C=0.5242, G=0.4758	0.00001
3	chr16:89985844	G/T	G	SNV	MC1R	exonic	1	c.178G>T	p.Val60Leu	missense	pathogenic		189	G	101	T	88	46,6	G=0.5344, T=0.4656	0.00001
4	chr16:23635348	A/C	A	SNV	PALB2	exonic	8	c.2816T>G	p.Leu939Trp	missense	probable-non-pathogenic		242	A	127	C	115	47,5	A=0.5248, C=0.4752	0.00001
4	chr16:23646857	A/G	A	SNV	PALB2	exonic	4	c.1010T>C	p.Leu337Ser	missense	probable-non-pathogenic		174	A	92	G	82	47,1	A=0.5287, G=0.4713	0.00001
7	chr11:108175463	A/T	A	SNV	ATM	exonic	37	c.5558A>T	p.Asp1853Val	missense		Pathogenic	628	A	299	T	329	52,4	A=0.4761, T=0.5239	0.00001
9	chr16:89985918	C/A	C	SNV	MC1R	exonic	1	c.252C>A	p.Asp84Glu	missense	other,pathogenic		292	C	150	A	142	48,6	C=0.5137, A=0.4863	0.00001
9	chr16:89986144	C/T	C	SNV	MC1R	exonic	1	c.478C>T	p.Arg160Trp	missense	pathogenic		306	C	153	T	153	50,0	C=0.5, T=0.5	0.00001
9	chr5:33951693	C/G	C	SNV	SLC45A2	exonic	5	c.1122G>C	p.Leu374Phe	missense			147	C	87	G	60	40,8	C=0.5918, G=0.4082	0.00001
10	chr3:70014091	G/A	G	SNV	MITF	exonic	10	c.1255G>A	p.Glu419Lys	missense			478	G	231	A	247	51,7	G=0.4833, A=0.5167	0.00001
10	chr11:89017961	G/A	G	SNV	TYR	exonic	4	c.1205G>A	p.Arg402Gln	missense	other,pathogenic		130	G	65	A	65	50,0	G=0.5, A=0.5	0.00001
11	chr9:21974760	C/T	C	SNV	CDKN2A	exonic	1	c.67G>A	p.Gly23Ser	missense		Pathogenic	122	C	64	T	58	47,5	C=0.5246, T=0.4754	0.00001
11	chr16:89985940	G/A	G	SNV	MC1R	exonic	1	c.274G>A	p.Val92Met	missense	pathogenic		426	G	251	A	175	41,1	G=0.5892, A=0.4108	0.00001
12	chr11:108175462	G/A	G	SNV	ATM	exonic	37	c.5557G>A	p.Asp1853Asn	missense		Pathogenic	653	G	313	A	340	52,1	G=0.4793, A=0.5207	0.00001
13	chr11:108175462	G/A	G	SNV	ATM	exonic	37	c.5557G>A	p.Asp1853Asn	missense		Pathogenic	411	G	214	A	197	47,9	G=0.5207, A=0.4793	0.00001
13	chr16:89986117	C/T	C	SNV	MC1R	exonic	1	c.451C>T	p.Arg151Cys	missense	other,pathogenic		365	C	173	T	192	52,6	C=0.474, T=0.526	0.00001
15	chr11:108175463	A/T	A	SNV	ATM	exonic	37	c.5558A>T	p.Asp1853Val	missense		Pathogenic	400	A	197	T	203	50,8	A=0.4925, T=0.5075	0.00001
15	chr16:89986546	G/C	G	SNV	MC1R	exonic	1	c.880G>C	p.Asp294His	missense	pathogenic		1860	G	903	C	957	51,5	G=0.4855, C=0.5145	0.00001
16	chr16:89985844	G/T	G	SNV	MC1R	exonic	1	c.178G>T	p.Val60Leu	missense	pathogenic		181	G	87	T	94	51,9	G=0.4807, T=0.5193	0.00001
16	chr11:89017961	G/A	G	SNV	TYR	exonic	4	c.1205G>A	p.Arg402Gln	missense	other,pathogenic		159	G	72	A	87	54,7	G=0.4528, A=0.5472	0.00001
20	chr16:23646191	T/C	T	SNV	PALB2	exonic	4	c.1676A>G	p.Gln559Arg	missense	probable-non-pathogenic		205	T	100	C	105	51,2	T=0.4878, C=0.5122	0.00001
21	chr11:108175462	G/A	G	SNV	ATM	exonic	37	c.5557G>A	p.Asp1853Asn	missense		Pathogenic	444	G	216	A	228	51,4	G=0.4865, A=0.5135	0.00001
21	chr9:21974760	C/T	C	SNV	CDKN2A	exonic	1	c.67G>A	p.Gly23Ser	missense		Pathogenic	238	C	166	T	72	30,3	C=0.6975, T=0.3025	0.00001
23	chr9:21974756	C/G	C	SNV	CDKN2A	exonic	1	c.71G>C	p.Arg24Pro	missense	other	Pathogenic	129	C	81	G	48	37,2	C=0.6279, G=0.3721	0.00001
23	chr16:89986117	C/T	C	SNV	MC1R	exonic	1	c.451C>T	p.Arg151Cys	missense	other,pathogenic		297	C	145	T	152	51,2	C=0.4882, T=0.5118	0.00001
23	chr16:89985940	G/A	G	SNV	MC1R	exonic	1	c.274G>A	p.Val92Met	missense	pathogenic		252	G	156	A	96	38,1	G=0.619, A=0.381	0.00001
23	chr16:23641461	C/G	C	SNV	PALB2	exonic	5	c.2014G>C	p.Glu672Gln	missense	probable-non-pathogenic		116	C	56	G	60	51,7	C=0.4828, G=0.5172	0.00001
23	chr7:124469334	G/A	G	SNV	POT1	exonic	16	c.1568C>T	p.Ser523Leu	missense			237	G	114	A	123	51,9	G=0.481, A=0.519	0.00001
25	chr16:89985844	G/T	G	SNV	MC1R	exonic	1	c.178G>T	p.Val60Leu	missense	pathogenic		119	G	59	T	60	50,4	G=0.4958, T=0.5042	0.00001
27	chr11:108175462	G/A	G	SNV	ATM	exonic	37	c.5557G>A	p.Asp1853Asn	missense		Pathogenic	681	G	351	A	330	48,5	G=0.5154, A=0.4846	0.00001
28	chr7:124475415	G/T	G	SNV	POT1	exonic	15	c.1423C>A	p.Pro475Thr	missense			322	G	154	T	168	52,2	G=0.4783, T=0.5217	0.00001
32	chr11:108175462	G/A	G	SNV	ATM	exonic	37	c.5557G>A	p.Asp1853Asn	missense		Pathogenic	184	G	97	A	87	47,3	G=0.5272, A=0.4728	0.00001
32	chr9:21970916	C/T	C	SNV	CDKN2A	exonic	2	c.442G>A	p.Ala148Thr	missense		Neutral	130	C	67	T	63	48,5	C=0.5154, T=0.4846	0.00001
33	chr16:89985940	G/A	G	SNV	MC1R	exonic	1	c.274G>A	p.Val92Met	missense	pathogenic		251	G	155	A	96	38,2	G=0.6175, A=0.3825	0.00001
33	chr16:23635370	C/T	C	SNV	PALB2	exonic	8	c.2794G>A	p.Val932Met	missense	probable-non-pathogenic		105	C	55	T	50	47,6	C=0.5238, T=0.4762	0.00001
34	chr11:108175462	G/A	G	SNV	ATM	exonic	37	c.5557G>A	p.Asp1853Asn	missense		Pathogenic	544	G	284	A	260	47,8	G=0.5221, A=0.4779	0.00001
34	chr9:21974756	C/G	C	SNV	CDKN2A	exonic	1	c.71G>C	p.Arg24Pro	missense	other	Pathogenic	133	C	65	G	68	51,1	C=0.4887, G=0.5113	0.00001
34	chr16:89986117	C/T	C	SNV	MC1R	exonic	1	c.451C>T	p.Arg151Cys	missense	other,pathogenic		710	C	354	T	356	50,1	C=0.4986, T=0.5014	0.00001
34	chr16:89986154	G/A	G	SNV	MC1R	exonic	1	c.488G>A	p.Arg163Gln	missense			404	G	203	A	201	49,8	G=0.5025, A=0.4975	0.00001
34	chr16:75690279	A/G	A	SNV	TERF2IP	exonic	3	c.970A>G	p.Lys324Glu	missense			185	A	97	G	88	47,6	A=0.5243, G=0.4757	0.00001
35	chr5:33951693	C/G	C	SNV	SLC45A2	exonic	5	c.1122G>C	p.Leu374Phe	missense			134	C	69	G	65	48,5	C=0.5149, G=0.4851	0.00001
36	chr11:108178702	G/C	G	SNV	ATM	exonic	38	c.5753G>C	p.Arg1918Thr	missense			184	G	94	C	90	48,9	G=0.5109, C=0.4891	0.00001
36	chr11:89017973	C/T	C	SNV	TYR	exonic	4	c.1217C>T	p.Pro406Leu	missense	pathogenic		126	C	79	T	47	37,3	C=0.627, T=0.373	0.00001
38	chr16:89985844	G/T	G	SNV	MC1R	exonic	1	c.178G>T	p.Val60Leu	missense	pathogenic		550	G	300	T	250	45,5	G=0.5455, T=0.4545	0.00001
38	chr7:124532326	C/T	C	SNV	POT1	exonic	6	c.118G>A	p.Gly40Arg	missense			691	C	586	T	105	15,2	C=0.848, T=0.152	0.00059
45	chr9:21974721	C/T	C	SNV	CDKN2A	exonic	1	c.106G>A	p.Ala36Thr	missense		Pathogenic	146	C	74	T	72	49,3	C=0.5068, T=0.4932	0.00001
45	chr16:89986117	C/T	C	SNV	MC1R	exonic	1	c.451C>T	p.Arg151Cys	missense	other,pathogenic		111	C	61	T	50	45,0	C=0.5495, T=0.4505	0.00001
47	chr7:124482969	A/C	A	SNV	POT1	exonic	13	c.1055T>G	p.Leu352Trp	missense			412	A	325	C	87	21,1	A=0.7888, C=0.2112	0.00001
49	chr16:89985844	T/T	G	SNV	MC1R	exonic	1	c.178G>T	p.Val60Leu	missense	pathogenic		964	G	28	T	936	97,1	G=0.029, T=0.971	0.00001
49	chr11:89017961	G/A	G	SNV	TYR	exonic	4	c.1205G>A	p.Arg402Gln	missense	other,pathogenic		269	G	131	A	138	51,3	G=0.487, A=0.513	0.00001
52	chr16:89986546	G/C	G	SNV	MC1R	exonic	1	c.880G>C	p.Asp294His	missense	pathogenic		726	G	395	C	331	45,6	G=0.5441, C=0.4559	0.00001
52	chr16:23641041	G/A	G	SNV	PALB2	exonic	5	c.2434C>T	p.Pro812Ser	missense	probable-pathogenic	Neutral	101	G	74	A	27	26,7	G=0.7327, A=0.2673	0.00911
53	chr16:89986091	G/A	G	SNV	MC1R	exonic	1	c.425G>A	p.Arg142His	missense			263	G	143	A	120	45,6	G=0.5437, A=0.4563	0.00001
53	chr16:23634293	C/T	C	SNV	PALB2	exonic	9	c.2993G>A	p.Gly998Glu	missense	probable-non-pathogenic		111	C	53	T	58	52,3	C=0.4775, T=0.5225	0.00001

Case No.	Locus	Genotype	Ref	Type	Gene	Location	Exon	Coding	Protein	Variant Effect	ClinVar	COSMIC PREDICTION	Coverage	Allele 1 Type	Allele 1 Coverage	Allele 2 Type	Allele 2 Coverage	% Mutated Allele 2	Allele Ratio	p-value
53	chr16:23646191	T/C	T	SNV	PALB2	exonic	4	c.1676A>G	p.Gln559Arg	missense	probable-non-pathogenic		127	T	64	C	63	49,6	T=0.5039, C=0.4961	0.00001
54	chr16:89985844	T/T	G	SNV	MC1R	exonic	1	c.178G>T	p.Val60Leu	missense	pathogenic		130	G	1	T	64	49,2	G=0.0154, T=0.9846	0.00001
56	chr9:21971179	G/A	G	SNV	CDKN2A	exonic	2	c.179C>T	p.Ala60Val	missense		Pathogenic	193	G	97	A	96	49,7	G=0.5026, A=0.4974	0.00001
56	chr16:89986117	C/T	C	SNV	MC1R	exonic	1	c.451C>T	p.Arg151Cys	missense	other,pathogenic		216	C	103	T	113	52,3	C=0.4769, T=0.5231	0.00001
56	chr16:89986091	G/A	G	SNV	MC1R	exonic	1	c.425G>A	p.Arg142His	missense			184	G	100	A	84	45,7	G=0.5435, A=0.4565	0.00001
60	chr16:89986144	C/T	C	SNV	MC1R	exonic	1	c.478C>T	p.Arg160Trp	missense	pathogenic		661	C	345	T	316	47,8	C=0.5219, T=0.4781	0.00001
60	chr11:89017961	G/A	G	SNV	TYR	exonic	4	c.1205G>A	p.Arg402Gln	missense	other,pathogenic		107	G	60	A	47	43,9	G=0.5607, A=0.4393	0.00001
61	chr11:108141872	T/TA	T	INDEL	ATM	exonic	19	c.2920_2921insA	p.Ser974fs	frameshiftInsertion			353	T	277	TA	76	21,5	T=0.7847, TA=0.2153	0.00001
61	chr16:89986122	C/A	C	SNV	MC1R	exonic	1	c.456C>A	p.Tyr152Ter	nonsense		Pathogenic	341	C	178	A	163	47,8	C=0.522, A=0.478	0.00001
61	chr7:124482969	A/C	A	SNV	POT1	exonic	13	c.1055T>G	p.Leu352Trp	missense			1222	A	977	C	245	20,0	A=0.7995, C=0.2005	0.00001
62	chr16:89986154	G/A	G	SNV	MC1R	exonic	1	c.488G>A	p.Arg163Gln	missense			230	G	110	A	120	52,2	G=0.4783, A=0.5217	0.00001
65	chr11:108218009	G/GT	G	INDEL	ATM	exonic	59	c.8588_8589insT	p.Tyr2864fs	frameshiftInsertion			212	G	138	GT	74	34,9	G=0.6509, GT=0.3491	0.00001
65	chr11:108141872	T/TA	T	INDEL	ATM	exonic	19	c.2920_2921insA	p.Ser974fs	frameshiftInsertion			643	T	529	TA	114	17,7	T=0.8227, TA=0.1773	0.00001
65	chr16:89985844	G/T	G	SNV	MC1R	exonic	1	c.178G>T	p.Val60Leu	missense	pathogenic		316	G	180	T	136	43,0	G=0.5696, T=0.4304	0.00001
65	chr16:89986091	G/A	G	SNV	MC1R	exonic	1	c.425G>A	p.Arg142His	missense			629	G	296	A	333	52,9	G=0.4706, A=0.5294	0.00001
66	chr11:108141872	T/TA	T	INDEL	ATM	exonic	19	c.2920_2921insA	p.Ser974fs	frameshiftInsertion			242	T	186	TA	56	23,1	T=0.7686, TA=0.2314	0.00001
66	chr9:21970916	C/T	C	SNV	CDKN2A	exonic	2	c.442G>A	p.Ala148Thr	missense		Neutral	187	C	97	T	90	48,1	C=0.5187, T=0.4813	0.00001
66	chr16:23646191	T/C	T	SNV	PALB2	exonic	4	c.1676A>G	p.Gln559Arg	missense	probable-non-pathogenic		154	T	83	C	71	46,1	T=0.539, C=0.461	0.00001
67	chr11:108141872	T/TA	T	INDEL	ATM	exonic	19	c.2920_2921insA	p.Ser974fs	frameshiftInsertion			298	T	254	TA	44	14,8	T=0.8523, TA=0.1477	0.04654
67	chr9:21970916	C/T	C	SNV	CDKN2A	exonic	2	c.442G>A	p.Ala148Thr	missense		Neutral	117	C	52	T	65	55,6	C=0.4444, T=0.5556	0.00001
67	chr16:89986117	C/T	C	SNV	MC1R	exonic	1	c.451C>T	p.Arg151Cys	missense	other,pathogenic		394	C	189	T	205	52,0	C=0.4797, T=0.5203	0.00001
68	chr11:108175462	G/A	G	SNV	ATM	exonic	37	c.5557G>A	p.Asp1853Asn	missense		Pathogenic	457	G	250	A	207	45,3	G=0.547, A=0.453	0.00001
68	chr16:89986117	C/T	C	SNV	MC1R	exonic	1	c.451C>T	p.Arg151Cys	missense	other,pathogenic		1294	C	615	T	679	52,5	C=0.4753, T=0.5247	0.00001
68	chr16:89985722	C/T	C	SNV	MC1R	exonic	1	c.56C>T	p.Thr19Ile	missense			283	C	136	T	147	51,9	C=0.4806, T=0.5194	0.00001
68	chr16:23646191	T/C	T	SNV	PALB2	exonic	4	c.1676A>G	p.Gln559Arg	missense	probable-non-pathogenic		295	T	136	C	159	53,9	T=0.461, C=0.539	0.00001
69	chr11:108141872	T/TA	T	INDEL	ATM	exonic	19	c.2920_2921insA	p.Ser974fs	frameshiftInsertion			439	T	376	TA	63	14,4	T=0.8565, TA=0.1435	0.0223
69	chr16:89985844	G/T	G	SNV	MC1R	exonic	1	c.178G>T	p.Val60Leu	missense	pathogenic		139	G	73	T	66	47,5	G=0.5252, T=0.4748	0.00001
73	chr16:89985940	G/A	G	SNV	MC1R	exonic	1	c.274G>A	p.Val92Met	missense	pathogenic		853	G	518	A	335	39,3	G=0.6073, A=0.3927	0.00001
73	chr7:124481185	C/A	C	SNV	POT1	exonic	14	c.1211G>T	p.Gly404Val	missense			863	C	422	A	441	51,1	C=0.489, A=0.511	0.00001
78	chr16:89985940	G/A	G	SNV	MC1R	exonic	1	c.274G>A	p.Val92Met	missense	pathogenic		512	G	310	A	202	39,5	G=0.6055, A=0.3945	0.00001
78	chr16:23646191	T/C	T	SNV	PALB2	exonic	4	c.1676A>G	p.Gln559Arg	missense	probable-non-pathogenic		251	T	145	C	106	42,2	T=0.5777, C=0.4223	0.00001
79	chr16:89985844	G/T	G	SNV	MC1R	exonic	1	c.178G>T	p.Val60Leu	missense	pathogenic		309	G	155	T	154	49,8	G=0.5016, T=0.4984	0.00001
79	chr16:89986144	C/T	C	SNV	MC1R	exonic	1	c.478C>T	p.Arg160Trp	missense	pathogenic		625	C	339	T	286	45,8	C=0.5424, T=0.4576	0.00001
80	chr9:21970916	C/T	C	SNV	CDKN2A	exonic	2	c.442G>A	p.Ala148Thr	missense		Neutral	1047	C	501	T	546	52,1	C=0.4785, T=0.5215	0.00001
80	chr16:89985844	G/T	G	SNV	MC1R	exonic	1	c.178G>T	p.Val60Leu	missense	pathogenic		404	G	209	T	195	48,3	G=0.5173, T=0.4827	0.00001
80	chr16:89986154	G/A	G	SNV	MC1R	exonic	1	c.488G>A	p.Arg163Gln	missense			1471	G	747	A	724	49,2	G=0.5078, A=0.4922	0.00001
80	chr16:75690279	A/G	A	SNV	TERF2IP	exonic	3	c.970A>G	p.Lys324Glu	missense			446	A	229	G	217	48,7	A=0.5135, G=0.4865	0.00001
80	chr11:89017961	G/A	G	SNV	TYR	exonic	4	c.1205G>A	p.Arg402Gln	missense	other,pathogenic		150	G	68	A	82	54,7	G=0.4533, A=0.5467	0.00001
81	chr11:108141872	T/TA	T	INDEL	ATM	exonic	19	c.2920_2921insA	p.Ser974fs	frameshiftInsertion			3884	T	3059	TA	825	21,2	T=0.7876, TA=0.2124	0.00001
81	chr16:89985844	T/T	G	SNV	MC1R	exonic	1	c.178G>T	p.Val60Leu	missense	pathogenic		1917	G	59	T	1858	96,9	G=0.0308, T=0.9692	0.00001
86	chr11:108124761	T/C	T	SNV	ATM	exonic	13	c.2119T>C	p.Ser707Pro	missense		Neutral	122	T	100	C	22	18,0	T=0.8197, C=0.1803	0.00636
86	chr11:108196152	AT/A	AT	INDEL	ATM	exonic	46	c.6689delT	p.Leu2231fs	frameshiftDeletion		None	367	AT	283	A	84	22,9	AT=0.7711, A=0.2289	0.00001
86	chr16:89986144	C/T	C	SNV	MC1R	exonic	1	c.478C>T	p.Arg160Trp	missense	pathogenic		1405	C	870	T	535	38,1	C=0.6192, T=0.3808	0.00001
86	chr16:23614812	CT/C	CT	INDEL	PALB2	exonic	13	c.3528delA	p.Asp1177fs	frameshiftDeletion			537	CT	172	C	365	68,0	CT=0.3203, C=0.6797	0.00001
86	chr16:23634303	CAA/CA	CAA	INDEL	PALB2	exonic	9	c.2982delT	p.Phe994fs	frameshiftDeletion			144	CA	99	CAA	40	27,8	CAA=0.2778, CA=0.6875, C=0.0347	0.00001
86	chr16:23614857	CA/C	CA	INDEL	PALB2	exonic	13	c.3483delT	p.Phe1161fs	frameshiftDeletion			298	CA	251	C	47	15,8	CA=0.8423, C=0.1577	0.02025
87	chr11:108150294	GA/G	GA	INDEL	ATM	exonic	23	c.3362delA	p.Asn1122fs	frameshiftDeletion			236	GA	178	G	58	24,6	GA=0.7542, G=0.2458	0.00003

Case No.	Locus	Genotype	Ref	Type	Gene	Location	Exon	Coding	Protein	Variant Effect	ClinVar	COSMIC PREDICTION	Coverage	Allele 1 Type	Allele 1 Coverage	Allele 2 Type	Allele 2 Coverage	% Mutated Allele 2	Allele Ratio	p-value
89	chr11:108196152	AT/A	AT	INDEL	ATM	exonic	46	c.6689delT	p.Leu2231fs	frameshiftDeletion		None	817	AT	654	A	163	20,0	AT=0.8005, A=0.1995	0.00001
89	chr3:52436850	A/G	A	SNV	BAP1	exonic	15	c.1928T>C	p.Ile643Thr	missense		Pathogenic	1026	A	252	G	774	75,4	A=0.2456, G=0.7544	0.00001
89	chr16:89985918	C/A	C	SNV	MC1R	exonic	1	c.252C>A	p.Asp84Glu	missense	other,pathogenic		2624	C	1359	A	1265	48,2	C=0.5179, A=0.4821	0.00001
91	chr3:52436850	A/G	A	SNV	BAP1	exonic	15	c.1928T>C	p.Ile643Thr	missense		Pathogenic	726	A	142	G	584	80,4	A=0.1956, G=0.8044	0.00001
92	chr11:108216476	CA/C	CA	INDEL	ATM	exonic	58	c.8426delA	p.Lys2811fs	frameshiftDeletion	probable-pathogenic		101	CA	71	C	30	29,7	CA=0.703, C=0.297	0.00097
92	chr3:52436850	A/G	A	SNV	BAP1	exonic	15	c.1928T>C	p.Ile643Thr	missense		Pathogenic	1074	A	231	G	843	78,5	A=0.2151, G=0.7849	0.00001
92	chr16:89986117	C/T	C	SNV	MC1R	exonic	1	c.451C>T	p.Arg151Cys	missense	other,pathogenic		3457	C	1706	T	1751	50,7	C=0.4935, T=0.5065	0.00001
92	chr16:89986144	C/T	C	SNV	MC1R	exonic	1	c.478C>T	p.Arg160Trp	missense	pathogenic		3346	C	1986	T	1360	40,6	C=0.5935, T=0.4065	0.00001
92	chr16:23647173	CT/C	CT	INDEL	PALB2	exonic	4	c.693delA	p.Gly232fs	frameshiftDeletion			172	CT	128	C	44	25,6	CT=0.7442, C=0.2558	0.00017
92	chr7:124493112	TA/T	TA	INDEL	POT1	exonic	10	c.782delT	p.Leu261Ter	nonsense			3802	TA	2632	T	1170	30,8	TA=0.6923, T=0.3077	0.00001
94	chr11:108121521	AC/A	AC	INDEL	ATM	exonic	10	c.1330delC	p.Gln445fs	frameshiftDeletion			197	AC	114	A	83	42,1	AC=0.5787, A=0.4213	0.00001
94	chr11:108150294	GA/G	GA	INDEL	ATM	exonic	23	c.3362delA	p.Asn1122fs	frameshiftDeletion			252	GA	193	G	59	23,4	GA=0.7659, G=0.2341	0.00002
94	chr3:52436850	A/G	A	SNV	BAP1	exonic	15	c.1928T>C	p.Ile643Thr	missense		Pathogenic	1199	A	264	G	935	78,0	A=0.2202, G=0.7798	0.00001
94	chr16:89985844	G/T	G	SNV	MC1R	exonic	1	c.178G>T	p.Val60Leu	missense	pathogenic		192	G	137	T	55	28,6	G=0.7135, T=0.2865	0.00001
94	chr16:23647173	CT/C	CT	INDEL	PALB2	exonic	4	c.693delA	p.Gly232fs	frameshiftDeletion			129	CT	103	C	26	20,2	CT=0.7984, C=0.2016	0.00951
96	chr11:108115627	C/T	C	SNV	ATM	exonic	7	c.775C>T	p.Leu259Phe	missense		Pathogenic	109	C	54	T	55	50,5	C=0.4954, T=0.5046	0.00001
96	chr3:52436850	A/G	A	SNV	BAP1	exonic	15	c.1928T>C	p.Ile643Thr	missense		Pathogenic	925	A	208	G	717	77,5	A=0.2249, G=0.7751	0.00001
96	chr16:89986546	G/C	G	SNV	MC1R	exonic	1	c.880G>C	p.Asp294His	missense	pathogenic		3993	G	1918	C	2075	52,0	G=0.4803, C=0.5197	0.00001
96	chr16:89986144	C/T	C	SNV	MC1R	exonic	1	c.478C>T	p.Arg160Trp	missense	pathogenic		3343	C	1998	T	1345	40,2	C=0.5977, T=0.4023	0.00001
96	chr16:23647173	CT/C	CT	INDEL	PALB2	exonic	4	c.693delA	p.Gly232fs	frameshiftDeletion			141	CT	90	CC	18	12,8	CT=0.6383, CC=0.1277, C=0.234	0.00001
98	chr11:108175462	G/A	G	SNV	ATM	exonic	37	c.5557G>A	p.Asp1853Asn	missense		Pathogenic	3287	G	1665	A	1622	49,3	G=0.5065, A=0.4935	0.00001
101	chr11:108160480	T/G	T	SNV	ATM	exonic	29	c.4388T>G	p.Phe1463Cys	missense		Pathogenic	138	T	63	G	75	54,3	T=0.4565, G=0.5435	0.00001
101	chr11:108123551	C/T	C	SNV	ATM	exonic	12	c.1810C>T	p.Pro604Ser	missense		Pathogenic	177	C	91	T	86	48,6	C=0.5141, T=0.4859	0.00001
101	chr3:52436850	A/G	A	SNV	BAP1	exonic	15	c.1928T>C	p.Ile643Thr	missense		Pathogenic	1366	A	292	G	1074	78,6	A=0.2138, G=0.7862	0.00001
102	chr11:108175462	G/A	G	SNV	ATM	exonic	37	c.5557G>A	p.Asp1853Asn	missense		Pathogenic	3976	G	2091	A	1745	43,9	G=0.5451, A=0.4549	0.00001
102	chr11:108124551	CA/C	CA	INDEL	ATM	exonic	13	c.1910delA	p.Asp639fs	frameshiftDeletion			271	CA	231	C	40	14,8	CA=0.8524, C=0.1476	0.02675
102	chr3:52436850	A/G	A	SNV	BAP1	exonic	15	c.1928T>C	p.Ile643Thr	missense		Pathogenic	1197	A	344	G	853	71,3	A=0.2874, G=0.7126	0.00001
102	chr16:89986154	G/A	G	SNV	MC1R	exonic	1	c.488G>A	p.Arg163Gln	missense			2196	G	1101	A	1095	49,9	G=0.5014, A=0.4986	0.00001
102	chr16:23632778	CA/C	CA	INDEL	PALB2	exonic	10	c.3017delT	p.Leu1006Ter	nonsense		Stop Codon	316	CA	151	C	165	52,2	CA=0.4778, C=0.5222	0.00001
102	chr16:23646687	GT/G	GT	INDEL	PALB2	exonic	4	c.1179delA	p.Lys393fs	frameshiftDeletion			132	GT	100	G	32	24,2	GT=0.7576, G=0.2424	0.00057
102	chr16:23614857	CA/C	CA	INDEL	PALB2	exonic	13	c.3483delT	p.Phe1161fs	frameshiftDeletion			476	CA	403	C	73	15,3	CA=0.8466, C=0.1534	0.01177
105	chr11:108175463	A/T	A	SNV	ATM	exonic	37	c.5558A>T	p.Asp1853Val	missense		Pathogenic	1728	A	904	T	824	47,7	A=0.5231, T=0.4769	0.00001
105	chr11:108196111	AT/A	AT	INDEL	ATM	exonic	46	c.6648delT	p.Phe2217fs	frameshiftDeletion			232	AT	151	A	81	34,9	AT=0.6509, A=0.3491	0.00001
105	chr3:52436850	A/G	A	SNV	BAP1	exonic	15	c.1928T>C	p.Ile643Thr	missense		Pathogenic	270	A	47	G	223	82,6	A=0.1741, G=0.8259	0.00001
106	chr3:52436850	A/G	A	SNV	BAP1	exonic	15	c.1928T>C	p.Ile643Thr	missense		Pathogenic	518	A	84	G	434	83,8	A=0.1622, G=0.8378	0.00001
106	chr16:75682028	A/C	A	SNV	KARS, TERF2IP	upstream, exonic	1	c.248A>C	p.Tyr83Ser	missense			221	A	186	C	35	15,8	A=0.8416, C=0.1584	0.00904
106	chr16:23635370	C/T	C	SNV	PALB2	exonic	8	c.2794G>A	p.Val932Met	missense	probable-non-pathogenic		480	C	286	T	194	40,4	C=0.5958, T=0.4042	0.00001
107	chr11:108175463	A/T	A	SNV	ATM	exonic	37	c.5558A>T	p.Asp1853Val	missense		Pathogenic	3987	A	2054	T	1933	48,5	A=0.5152, T=0.4848	0.00001
107	chr11:108143522	AAT/AA	AAT	INDEL	ATM	exonic	22	c.3229delT	p.Leu1078fs	frameshiftDeletion			120	AAT	89	AA	28	23,3	AAT=0.7417, AA=0.2333, A=0.025	0.00106
107	chr11:108124551	CA/C	CA	INDEL	ATM	exonic	13	c.1910delA	p.Asp639fs	frameshiftDeletion			156	CA	130	C	26	16,7	CA=0.8333, C=0.1667	0.0142
107	chr16:89986531	T/C	T	SNV	MC1R	exonic	1	c.865T>C	p.Cys289Arg	missense			3997	T	2062	C	1935	48,4	T=0.5159, C=0.4841	0.00001
107	chr16:89986154	G/A	G	SNV	MC1R	exonic	1	c.488G>A	p.Arg163Gln	missense			1953	G	1020	A	933	47,8	G=0.5223, A=0.4777	0.00001
107	chr16:23634303	CA/C	CA	INDEL	PALB2	exonic	9	c.2982delT	p.Phe994fs	frameshiftDeletion			136	CA	52	C	84	61,8	CA=0.3824, C=0.6176	0.00001
109	chr11:108196152	AT/A	AT	INDEL	ATM	exonic	46	c.6689delT	p.Leu2231fs	frameshiftDeletion		None	708	AT	545	A	163	23,0	AT=0.7698, A=0.2302	0.00001

Case No.	Locus	Genotype	Ref	Type	Gene	Location	Exon	Coding	Protein	Variant Effect	ClinVar	COSMIC PREDICTION	Coverage	Allele 1 Type	Allele 1 Coverage	Allele 2 Type	Allele 2 Coverage	% Mutated Allele 2	Allele Ratio	p-value
109	chr11:108126978	CT/C	CT	INDEL	ATM	exonic	14	c.2162delT	p.Leu722fs	frameshiftDeletion			564	CT	402	C	162	28,7	CT=0.7128, C=0.2872	0.00001
109	chr11:108124551	CA/C	CA	INDEL	ATM	exonic	13	c.1910delA	p.Asp639fs	frameshiftDeletion			332	CA	270	C	62	18,7	CA=0.8133, C=0.1867	0.00055
109	chr3:52436850	A/G	A	SNV	BAP1	exonic	15	c.1928T>C	p.Ile643Thr	missense		Pathogenic	986	A	278	G	708	71,8	A=0.2819, G=0.7181	0.00001
109	chr16:89986117	C/T	C	SNV	MC1R	exonic	1	c.451C>T	p.Arg151Cys	missense	other,pathogenic		2749	C	1519	T	1230	44,7	C=0.5526, T=0.4474	0.00001
109	chr16:23632778	CA/C	CA	INDEL	PALB2	exonic	10	c.3017delT	p.Leu1006Ter	nonsense		Stop Codon	305	CA	122	C	183	60,0	CA=0.4, C=0.6	0.00001
109	chr16:23634303	CA/C	CA	INDEL	PALB2	exonic	9	c.2982delT	p.Phe994fs	frameshiftDeletion			145	CA	62	C	83	57,2	CA=0.4276, C=0.5724	0.00001
109	chr7:124469337	GT/G	GT	INDEL	POT1	exonic	16	c.1564delA	p.Thr522fs	frameshiftDeletion			135	GT	86	G	49	36,3	GT=0.637, G=0.363	0.00001
111	chr11:108175462	G/A	G	SNV	ATM	exonic	37	c.5557G>A	p.Asp1853Asn	missense		Pathogenic	2974	G	1555	A	1339	45,0	G=0.5373, A=0.4627	0.00001
111	chr11:108124551	CA/C	CA	INDEL	ATM	exonic	13	c.1910delA	p.Asp639fs	frameshiftDeletion			150	CA	102	C	48	32,0	CA=0.68, C=0.32	0.00001
111	chr3:52436850	A/G	A	SNV	BAP1	exonic	15	c.1928T>C	p.Ile643Thr	missense		Pathogenic	507	A	132	G	375	74,0	A=0.2604, G=0.7396	0.00001
111	chr16:23646191	T/C	T	SNV	PALB2	exonic	4	c.1676A>G	p.Gln559Arg	missense	probable-non-pathogenic		144	T	74	C	70	48,6	T=0.5139, C=0.4861	0.00001
111	chr16:23641461	C/G	C	SNV	PALB2	exonic	5	c.2014G>C	p.Glu672Gln	missense	probable-non-pathogenic		129	C	71	G	58	45,0	C=0.5504, G=0.4496	0.00001
111	chr16:23634293	C/T	C	SNV	PALB2	exonic	9	c.2993G>A	p.Gly998Glu	missense	probable-non-pathogenic		108	C	65	T	43	39,8	C=0.6019, T=0.3981	0.00001
111	chr16:23634303	CA/C	CA	INDEL	PALB2	exonic	9	c.2982delT	p.Phe994fs	frameshiftDeletion			117	CA	42	C	75	64,1	CA=0.359, C=0.641	0.00001
112	chr11:108124551	CA/C	CA	INDEL	ATM	exonic	13	c.1910delA	p.Asp639fs	frameshiftDeletion			153	CA	113	C	40	26,1	CA=0.7386, C=0.2614	0.00076
112	chr16:89986144	C/T	C	SNV	MC1R	exonic	1	c.478C>T	p.Arg160Trp	missense	pathogenic		1255	C	688	T	567	45,2	C=0.5482, T=0.4518	0.00001
112	chr16:23632778	CA/C	CA	INDEL	PALB2	exonic	10	c.3017delT	p.Leu1006Ter	nonsense		Stop Codon	186	CA	59	C	127	68,3	CA=0.3172, C=0.6828	0.00001
112	chr16:23646191	T/C	T	SNV	PALB2	exonic	4	c.1676A>G	p.Gln559Arg	missense	probable-non-pathogenic		143	T	68	C	75	52,4	T=0.4755, C=0.5245	0.00001
112	chr16:23641461	C/G	C	SNV	PALB2	exonic	5	c.2014G>C	p.Glu672Gln	missense	probable-non-pathogenic		144	C	82	G	62	43,1	C=0.5694, G=0.4306	0.00001
112	chr16:23614812	CT/C	CT	INDEL	PALB2	exonic	13	c.3528delA	p.Asp1177fs	frameshiftDeletion			381	CT	107	C	274	71,9	CT=0.2808, C=0.7192	0.00001
112	chr16:23614857	CA/C	CA	INDEL	PALB2	exonic	13	c.3483delT	p.Phe1161fs	frameshiftDeletion			240	CA	197	C	43	17,9	CA=0.8208, C=0.1792	0.00358
112	chr7:124503493	AT/A	AT	INDEL	POT1	exonic	8	c.456delA	p.Lys152fs	frameshiftDeletion			154	AT	116	A	38	24,7	AT=0.7532, A=0.2468	0.00472
116	chr11:108202221	AATG/ATG	ATTG	SNV,INDEL	ATM	exonic, exonic	51	c.7567T>A, c.7567delT	p.Leu2523Met, p.Leu2523fs	missense, frameshiftDeletion	probable-pathogenic		318	AATG	98	ATG	220	69,2	ATTG=0.0, AATG=0.3082, AT=0.0, ATG=0.6918	0.00001
116	chr16:89985940	G/A	G	SNV	MC1R	exonic	1	c.274G>A	p.Val92Met	missense	pathogenic		908	G	472	A	436	48,0	G=0.5198, A=0.4802	0.00001
117	chr11:108196152	AT/A	AT	INDEL	ATM	exonic	46	c.6689delT	p.Leu2231fs	frameshiftDeletion		None	369	AT	286	A	83	22,5	AT=0.7751, A=0.2249	0.00001
117	chr16:89985940	G/A	G	SNV	MC1R	exonic	1	c.274G>A	p.Val92Met	missense	pathogenic		1459	G	820	A	639	43,8	G=0.562, A=0.438	0.00001
117	chr16:23632778	CAA/CA	CAA	INDEL	PALB2	exonic	10	c.3017delT	p.Leu1006Ter	nonsense		Stop Codon	173	CAA	61	CA	103	59,5	CAA=0.3526, CA=0.5954, C=0.052	0.00001
118	chr11:108216476	CA/C	CA	INDEL	ATM	exonic	58	c.8426delA	p.Lys2811fs	frameshiftDeletion	probable-pathogenic		101	CA	68	C	33	32,7	CA=0.6733, C=0.3267	0.00003
118	chr11:108122700	T/C	T	SNV	ATM	exonic	11	c.1744T>C	p.Phe582Leu	missense			373	T	182	C	191	51,2	T=0.4879, C=0.5121	0.00001
118	chr11:108142090	A/G	A	SNV	ATM	exonic	20	c.3034A>G	p.Arg1012Gly	missense			107	A	87	G	20	18,7	A=0.8131, G=0.1869	0.00591
118	chr16:89986117	C/T	C	SNV	MC1R	exonic	1	c.451C>T	p.Arg151Cys	missense	other,pathogenic		1826	C	980	T	846	46,3	C=0.5367, T=0.4633	0.00001
119	chr3:52436850	A/G	A	SNV	BAP1	exonic	15	c.1928T>C	p.Ile643Thr	missense		Pathogenic	1002	A	272	G	730	72,9	A=0.2715, G=0.7285	0.00001
119	chr16:23632778	CA/C	CA	INDEL	PALB2	exonic	10	c.3017delT	p.Leu1006Ter	nonsense		Stop Codon	309	CA	170	C	139	45,0	CA=0.5502, C=0.4498	0.00001
119	chr16:23634303	CA/C	CA	INDEL	PALB2	exonic	9	c.2982delT	p.Phe994fs	frameshiftDeletion			232	CA	117	C	115	49,6	CA=0.5043, C=0.4957	0.00001
120	chr11:108202221	AATG/ATG	ATTG	SNV, INDEL	ATM	exonic, exonic	51	c.7567T>A, c.7567delT	p.Leu2523Met, p.Leu2523fs	missense, frameshiftDeletion	probable-pathogenic		307	ATG	212	AATG	94	30,6	ATTG=0.0033, AATG=0.3062, ATG=0.6906	0.00001
120	chr9:21971179	G/A	G	SNV	CDKN2A	exonic	2	c.179C>T	p.Ala60Val	missense		Pathogenic	242	G	147	A	95	39,3	G=0.6074, A=0.3926	0.00001
120	chr16:89985940	G/A	G	SNV	MC1R	exonic	1	c.274G>A	p.Val92Met	missense	pathogenic		612	G	338	A	274	44,8	G=0.5523, A=0.4477	0.00001
120	chr16:23646191	T/C	T	SNV	PALB2	exonic	4	c.1676A>G	p.Gln559Arg	missense	probable-non-pathogenic		120	T	76	C	44	36,7	T=0.6333, C=0.3667	0.00001
122	chr16:89986144	C/T	C	SNV	MC1R	exonic	1	c.478C>T	p.Arg160Trp	missense	pathogenic		3623	C	2112	T	1511	41,7	C=0.5829, T=0.4171	0.00001

Case No.	Locus	Genotype	Ref	Type	Gene	Location	Exon	Coding	Protein	Variant Effect	ClinVar	COSMIC PREDICTION	Coverage	Allele 1 Type	Allele 1 Coverage	Allele 2 Type	Allele 2 Coverage	% Mutated Allele 2	Allele Ratio	p-value
122	chr16:89986546	G/C	G	SNV	MC1R	exonic	1	c.880G>C	p.Asp294His	missense	pathogenic		3987	G	1955	C	2032	51,0	G=0.4903, C=0.5097	0.00001
122	chr16:23646191	T/C	T	SNV	PALB2	exonic	4	c.1676A>G	p.Gln559Arg	missense	probable-non-pathogenic		327	T	175	C	152	46,5	T=0.5352, C=0.4648	0.00001
123	chr16:89986144	C/T	C	SNV	MC1R	exonic	1	c.478C>T	p.Arg160Trp	missense	pathogenic		1781	C	1038	T	743	41,7	C=0.5828, T=0.4172	0.00001
123	chr16:23646191	T/C	T	SNV	PALB2	exonic	4	c.1676A>G	p.Gln559Arg	missense	probable-non-pathogenic		288	T	137	C	151	52,4	T=0.4757, C=0.5243	0.00001
123	chr16:23641461	C/G	C	SNV	PALB2	exonic	5	c.2014G>C	p.Glu672Gln	missense	probable-non-pathogenic		767	C	385	G	382	49,8	C=0.502, G=0.498	0.00001
126	chr11:108196152	AT/A	AT	INDEL	ATM	exonic	46	c.6689delT	p.Leu2231fs	frameshiftDeletion		None	308	AT	255	A	53	17,2	AT=0.8279, A=0.1721	0.0004
126	chr11:108175462	G/A	G	SNV	ATM	exonic	37	c.5557G>A	p.Asp1853Asn	missense		Pathogenic	2470	G	1327	A	1006	40,7	G=0.5688, A=0.4312	0.00001
126	chr16:89986117	C/T	C	SNV	MC1R	exonic	1	c.451C>T	p.Arg151Cys	missense	other,pathogenic		1123	C	604	T	519	46,2	C=0.5378, T=0.4622	0.00001
126	chr16:23634384	CT/C	CT	INDEL	PALB2	exonic	9	c.2901delA	p.Ala968fs	frameshiftDeletion			151	CT	113	C	38	25,2	CT=0.7483, C=0.2517	0.00059
127	chr11:108175462	G/A	G	SNV	ATM	exonic	37	c.5557G>A	p.Asp1853Asn	missense		Pathogenic	2330	G	1232	A	1098	47,1	G=0.5288, A=0.4712	0.00001
127	chr3:52436850	A/G	A	SNV	BAP1	exonic	15	c.1928T>C	p.Ile643Thr	missense		Pathogenic	422	A	121	G	301	71,3	A=0.2867, G=0.7133	0.00001
128	chr16:89986117	C/T	C	SNV	MC1R	exonic	1	c.451C>T	p.Arg151Cys	missense	other,pathogenic		1547	C	821	T	726	46,9	C=0.5307, T=0.4693	0.00001
128	chr16:89986091	G/A	G	SNV	MC1R	exonic	1	c.425G>A	p.Arg142His	missense			629	G	338	A	291	46,3	G=0.5374, A=0.4626	0.00001
129	chr16:23646191	T/C	T	SNV	PALB2	exonic	4	c.1676A>G	p.Gln559Arg	missense	probable-non-pathogenic		174	T	91	C	83	47,7	T=0.523, C=0.477	0.00001
130	chr11:108138003	T/C	T	SNV	ATM	exonic	17	c.2572T>C	p.Phe858Leu	missense		Pathogenic	150	T	87	C	63	42,0	T=0.58, C=0.42	0.00001
132	chr11:108196152	AT/A	AT	INDEL	ATM	exonic	46	c.6689delT	p.Leu2231fs	frameshiftDeletion		None	694	AT	596	A	98	14,1	AT=0.8588, A=0.1412	0.00255
132	chr11:108175462	G/A	G	SNV	ATM	exonic	37	c.5557G>A	p.Asp1853Asn	missense		Pathogenic	3958	G	2112	A	1644	41,5	G=0.5623, A=0.4377	0.00001
132	chr11:108150294	GA/G	GA	INDEL	ATM	exonic	23	c.3362delA	p.Asn1122fs	frameshiftDeletion			126	GA	91	G	35	27,8	GA=0.7222, G=0.2778	0.00006
132	chr11:108170508	TA/T	TA	INDEL	ATM	exonic	34	c.5074delA	p.Cys1726fs	frameshiftDeletion			125	TA	99	T	26	20,8	TA=0.792, T=0.208	0.0106
132	chr3:52436850	A/G	A	SNV	BAP1	exonic	15	c.1928T>C	p.Ile643Thr	missense		Pathogenic	1046	A	349	G	697	66,6	A=0.3337, G=0.6663	0.00001
132	chr16:89986546	G/C	G	SNV	MC1R	exonic	1	c.880G>C	p.Asp294His	missense	pathogenic		3984	G	1961	C	2023	50,8	G=0.4922, C=0.5078	0.00001
132	chr16:23634303	CA/C	CA	INDEL	PALB2	exonic	9	c.2982delT	p.Phe994fs	frameshiftDeletion			171	CA	92	C	79	46,2	CA=0.538, C=0.462	0.00001
132	chr16:23641767	CT/C	CT	INDEL	PALB2	exonic	5	c.1707delA	p.Glu570fs	frameshiftDeletion			188	CT	142	C	46	24,5	CT=0.7553, C=0.2447	0.00018
132	chr7:124503677	CT/C	CT	INDEL	POT1	exonic	8	c.272delA	p.Lys91fs	frameshiftDeletion			511	CT	428	C	83	16,2	CT=0.8376, C=0.1624	0.00048
135	chr11:108196152	AT/A	AT	INDEL	ATM	exonic	46	c.6689delT	p.Leu2231fs	frameshiftDeletion		None	568	AT	451	A	117	20,6	AT=0.794, A=0.206	0.00001
135	chr3:52436850	A/G	A	SNV	BAP1	exonic	15	c.1928T>C	p.Ile643Thr	missense		Pathogenic	1075	A	283	G	792	73,7	A=0.2633, G=0.7367	0.00001
135	chr9:21971120	G/A	G	SNV	CDKN2A	exonic	2	c.238C>T	p.Arg80Ter	nonsense	pathogenic		816	G	550	A	266	32,6	G=0.6740, AG=0.3260	0.00001
135	chr16:89986546	G/C	G	SNV	MC1R	exonic	1	c.880G>C	p.Asp294His	missense	pathogenic		3990	G	1865	C	2125	53,3	G=0.4674, C=0.5326	0.00001
135	chr16:23614812	CT/C	CT	INDEL	PALB2	exonic	13	c.3528delA	p.Asp1177fs	frameshiftDeletion			572	CT	196	C	376	65,7	CT=0.3427, C=0.6573	0.00001
135	chr16:23646687	GT/G	GT	INDEL	PALB2	exonic	4	c.1179delA	p.Lys393fs	frameshiftDeletion			108	GT	69	G	39	36,1	GT=0.6389, G=0.3611	0.00001
135	chr16:23634384	CT/C	CT	INDEL	PALB2	exonic	9	c.2901delA	p.Ala968fs	frameshiftDeletion			336	CT	275	C	61	18,2	CT=0.8185, C=0.1815	0.00214
135	chr7:124503677	CT/C	CT	INDEL	POT1	exonic	8	c.272delA	p.Lys91fs	frameshiftDeletion			398	CT	338	C	60	15,1	CT=0.8492, C=0.1508	0.01461
136	chr11:108175462	G/A	G	SNV	ATM	exonic	37	c.5557G>A	p.Asp1853Asn	missense		Pathogenic	2476	G	1263	A	1098	44,3	G=0.5349, A=0.4651	0.00001
136	chr11:108126978	CT/C	CT	INDEL	ATM	exonic	14	c.2162delT	p.Leu722fs	frameshiftDeletion			203	CT	148	C	55	27,1	CT=0.7291, C=0.2709	0.00004
136	chr11:108122627	G/T	G	SNV	ATM	exonic	11	c.1671G>T	p.Met557Ile	missense			373	G	313	T	60	16,1	G=0.8391, T=0.1609	0.00573
136	chr16:89985940	G/A	G	SNV	MC1R	exonic	1	c.274G>A	p.Val92Met	missense	pathogenic		1205	G	670	A	535	44,4	G=0.556, A=0.444	0.00001
136	chr16:23634303	CA/C	CA	INDEL	PALB2	exonic	9	c.2982delT	p.Phe994fs	frameshiftDeletion			107	CA	43	C	64	59,8	CA=0.4019, C=0.5981	0.00001
138	chr11:108124551	CA/C	CA	INDEL	ATM	exonic	13	c.1910delA	p.Asp639fs	frameshiftDeletion			133	CA	96	C	37	27,8	CA=0.7218, C=0.2782	0.00049
138	chr3:52436850	A/G	A	SNV	BAP1	exonic	15	c.1928T>C	p.Ile643Thr	missense		Pathogenic	757	A	230	G	527	69,6	A=0.3038, G=0.6962	0.00001
138	chr16:89986546	G/C	G	SNV	MC1R	exonic	1	c.880G>C	p.Asp294His	missense	pathogenic		3993	G	1974	C	2019	50,6	G=0.4944, C=0.5056	0.00001
138	chr16:23641767	CT/C	CT	INDEL	PALB2	exonic	5	c.1707delA	p.Glu570fs	frameshiftDeletion			110	CT	72	C	38	34,5	CT=0.6545, C=0.3455	0.00001

Case No.	Locus	Genotype	Ref	Type	Gene	Location	Exon	Coding	Protein	Variant Effect	ClinVar	COSMIC PREDICTION	Coverage	Allele 1 Type	Allele 1 Coverage	Allele 2 Type	Allele 2 Coverage	% Mutated Allele 2	Allele Ratio	p-value
138	chr7:124503677	CT/C	CT	INDEL	POT1	exonic	8	c.272delA	p.Lys91fs	frameshiftDeletion			421	CT	319	C	102	24,2	CT=0.7577, C=0.2423	0.00001
**141**	**chr11:108216476**	**CA/C**	**CA**	**INDEL**	**ATM**	**exonic**	**58**	**c.8426delA**	**p.Lys2811fs**	**frameshiftDeletion**	**probable-pathogenic**		**103**	**CA**	**73**	**C**	**30**	**29,1**	**CA=0.7087, C=0.2913**	**0.00073**
**141**	**chr3:52436850**	**A/G**	**A**	**SNV**	**BAP1**	**exonic**	**15**	**c.1928T>C**	**p.Ile643Thr**	**missense**		**Pathogenic**	**1159**	**A**	**253**	**G**	**906**	**78,2**	**A=0.2183, G=0.7817**	**0.00001**
**141**	**chr16:89986117**	**C/T**	**C**	**SNV**	**MC1R**	**exonic**	**1**	**c.451C>T**	**p.Arg151Cys**	**missense**	**other,pathogenic**		**3986**	**C**	**2181**	**T**	**1805**	**45,3**	**C=0.5472, T=0.4528**	**0.00001**
141	chr16:23646191	T/C	T	SNV	PALB2	exonic	4	c.1676A>G	p.Gln559Arg	missense	probable-non-pathogenic		346	T	177	C	169	48,8	T=0.5116, C=0.4884	0.00001
142	chr11:108124551	CA/C	CA	INDEL	ATM	exonic	13	c.1910delA	p.Asp639fs	frameshiftDeletion			316	CA	252	C	64	20,3	CA=0.7975, C=0.2025	0.00085
**142**	**chr3:52436850**	**A/G**	**A**	**SNV**	**BAP1**	**exonic**	**15**	**c.1928T>C**	**p.Ile643Thr**	**missense**		**Pathogenic**	**1045**	**A**	**253**	**G**	**792**	**75,8**	**A=0.2421, G=0.7579**	**0.00001**
**142**	**chr16:89986546**	**G/C**	**G**	**SNV**	**MC1R**	**exonic**	**1**	**c.880G>C**	**p.Asp294His**	**missense**	**pathogenic**		**3992**	**G**	**2026**	**C**	**1966**	**49,2**	**G=0.5075, C=0.4925**	**0.00001**
142	chr16:23641767	CT/C	CT	INDEL	PALB2	exonic	5	c.1707delA	p.Glu570fs	frameshiftDeletion			145	CT	100	C	45	31,0	CT=0.6897, C=0.3103	0.00002
143	chr11:108202222	T/A	T	SNV	ATM	exonic	51	c.7567T>A	p.Leu2523Met	missense			217	T	179	A	38	17,5	T=0.8249, A=0.1751	0.00477
143	chr16:89986154	G/A	G	SNV	MC1R	exonic	1	c.488G>A	p.Arg163Gln	missense			489	G	244	A	245	50,1	G=0.499, A=0.501	0.00001
145	chr11:108196152	AT/A	AT	INDEL	ATM	exonic	46	c.6689delT	p.Leu2231fs	frameshiftDeletion		None	536	AT	413	A	123	22,9	AT=0.7705, A=0.2295	0.00001
145	chr11:108124551	CA/C	CA	INDEL	ATM	exonic	13	c.1910delA	p.Asp639fs	frameshiftDeletion			263	CA	209	C	54	20,5	CA=0.7947, C=0.2053	0.00036
**145**	**chr11:108143456**	**C/G**	**C**	**SNV**	**ATM**	**exonic**	**22**	**c.3161C>G**	**p.Pro1054Arg**	**missense**		**Pathogenic**	**102**	**C**	**73**	**G**	**29**	**28,4**	**C=0.7157, G=0.2843**	**0.00001**
145	chr16:23637613	A/C	A	SNV	PALB2	exonic	7	c.2692T>G	p.Trp898Gly	missense			189	A	156	C	33	17,5	A=0.8254, C=0.1746	0.00349
146	chr11:108150294	GA/G	GA	INDEL	ATM	exonic	23	c.3362delA	p.Asn1122fs	frameshiftDeletion			266	GA	219	G	47	17,7	GA=0.8233, G=0.1767	0.00361
146	chr16:23646191	T/C	T	SNV	PALB2	exonic	4	c.1676A>G	p.Gln559Arg	missense	probable-non-pathogenic		249	T	119	C	130	52,2	T=0.4779, C=0.5221	0.00001
146	chr16:23641461	C/G	C	SNV	PALB2	exonic	5	c.2014G>C	p.Glu672Gln	missense	probable-non-pathogenic		784	C	405	G	379	48,3	C=0.5166, G=0.4834	0.00001
**149**	**chr11:108175462**	**G/A**	**G**	**SNV**	**ATM**	**exonic**	**37**	**c.5557G>A**	**p.Asp1853Asn**	**missense**		**Pathogenic**	**3967**	**G**	**2079**	**A**	**1888**	**47,6**	**G=0.5241, A=0.4759**	**0.00001**
**149**	**chr16:89985844**	**G/T**	**G**	**SNV**	**MC1R**	**exonic**	**1**	**c.178G>T**	**p.Val60Leu**	**missense**	**pathogenic**		**168**	**G**	**111**	**T**	**57**	**33,9**	**G=0.6607, T=0.3393**	**0.00001**
**150**	**chr3:52436850**	**A/G**	**A**	**SNV**	**BAP1**	**exonic**	**15**	**c.1928T>C**	**p.Ile643Thr**	**missense**		**Pathogenic**	**967**	**A**	**356**	**G**	**611**	**63,2**	**A=0.3681, G=0.6319**	**0.00001**
**151**	**chr11:108160480**	**T/G**	**T**	**SNV**	**ATM**	**exonic**	**29**	**c.4388T>G**	**p.Phe1463Cys**	**missense**		**Pathogenic**	**267**	**T**	**156**	**G**	**111**	**41,6**	**T=0.5843, G=0.4157**	**0.00001**
**152**	**chr16:89986546**	**G/C**	**G**	**SNV**	**MC1R**	**exonic**	**1**	**c.880G>C**	**p.Asp294His**	**missense**	**pathogenic**		**2876**	**G**	**1389**	**C**	**1487**	**51,7**	**G=0.4830, C=0.5170**	**0.00001**
152	chr16:89986091	G/A	G	SNV	MC1R	exonic	1	c.425G>A	p.Arg142His	missense			580	G	393	A	187	32,2	G=0.6776, A=0.3224	0.00001
**155**	**chr16:89986144**	**C/T**	**C**	**SNV**	**MC1R**	**exonic**	**1**	**c.478C>T**	**p.Arg160Trp**	**missense**	**pathogenic**		**1839**	**C**	**1089**	**T**	**750**	**40,8**	**C=0.5922, T=0.4078**	**0.00001**
155	chr7:124469337	GT/G	GT	INDEL	POT1	exonic	16	c.1564delA	p.Thr522fs	frameshiftDeletion			144	GT	92	G	52	36,1	GT=0.6389, G=0.3611	0.00001
**157**	**chr3:52436850**	**A/G**	**A**	**SNV**	**BAP1**	**exonic**	**15**	**c.1928T>C**	**p.Ile643Thr**	**missense**		**Pathogenic**	**887**	**A**	**393**	**G**	**494**	**55,7**	**A=0.4431, G=0.5569**	**0.00001**
**157**	**chr16:89986546**	**G/C**	**G**	**SNV**	**MC1R**	**exonic**	**1**	**c.880G>C**	**p.Asp294His**	**missense**	**pathogenic**		**1962**	**G**	**956**	**C**	**1006**	**51,3**	**G=0.4873, C=0.5127**	**0.00001**
**159**	**chr3:52436850**	**A/G**	**A**	**SNV**	**BAP1**	**exonic**	**15**	**c.1928T>C**	**p.Ile643Thr**	**missense**		**Pathogenic**	**525**	**A**	**82**	**G**	**443**	**84,4**	**A=0.1562, G=0.8438**	**0.00001**
160	chr11:108202222	T/A	T	SNV	ATM	exonic	51	c.7567T>A	p.Leu2523Met	missense			302	T	236	A	66	21,9	T=0.7815, A=0.2185	0.00038
**160**	**chr16:23632778**	**CA/C**	**CA**	**INDEL**	**PALB2**	**exonic**	**10**	**c.3017delT**	**p.Leu1006Ter**	**nonsense**		**Stop Codon**	**411**	**CA**	**279**	**C**	**132**	**32,1**	**CA=0.6788, C=0.3212**	**0.00001**
