# Supplementary material for: Germline and somatic mutations in patients with multiple primary melanomas: a next generation sequencing study
Source: BMC Cancer. 2019 Aug 5;19:772. doi: 10.1186/s12885-019-5984-7 (PMC6683413; doi:10.1186/s12885-019-5984-7)
Supplement: Supplementary file 2 — Table S2. The 73 somatic variants found in our study, in detail. In bold, variants classified as pathogenic/likely pathogenic mutations. (PDF 76 kb) [file 12885_2019_5984_MOESM2_ESM.pdf]

Case #	Sample	Locus	Genotype	Ref	Type	Gene	Location	Exon	Coding	Protein	Variant effect	Clinvar/COSMIC	Coverage	Allele coverage	Mutated allele	Mutated allele %	pvalue
23	MM1	chr9:21974758	ACCCC/ACTCC	ACCCC	SNV	CDKN2A	exonic	1	c.67G>A	p.Gly23Ser	missense	Likely pathogenic	423	ACCCC=224, ACCCT=14, ACTCC=50	64	15,1	0.00001
23	MM1	chr3:178927410	A/G	A	SNV	PIK3CA	exonic	7	c.1173A>G	p.Ile391Met	missense	Likely benign,not provided	916	A=514, G=402	402	43,9	0.00001
23	MM1	chr17:7579391	G/A	G	SNV	TP53	exonic	4	c.296C>T	p.Ser99Phe	missense		360	G=342, A=18	18	5,0	0.00019
23	MM2	chr9:21974758	ACCCC/ACTCC	ACCCC	SNV	CDKN2A	exonic	1	c.67G>A	p.Gly23Ser	missense	Likely pathogenic	456	ACCCC=396, ACCCT=7, ACTCC=53	60	13,2	0.00001
23	MM2	chr17:7577082	C/T	C	SNV	TP53	exonic	8	c.856G>A	p.Glu286Lys	missense	Pathogenic/Likely pathogenic	322	C=306, T=16	16	5,0	0.00042
23	MM3	chr9:21974758	ACCCC/ACTCC	ACCCC	SNV	CDKN2A	exonic	1	c.67G>A	p.Gly23Ser	missense	Likely pathogenic	387	ACCCC=331, ACCCT=9, ACTCC=47	47	12,1	0.00001
23	MM3	chr17:7579472	G/C	G	SNV	TP53	exonic	4	c.215C>G	p.Pro72Arg	missense	Benign,Uncertain significance	353	G=15, C=324	324	91,8	0.00001
23	MM4	chr9:21974758	ACCCC/ACTCC	ACCCC	SNV	CDKN2A	exonic	1	c.67G>A	p.Gly23Ser	missense	Likely pathogenic	421	ACCCC=367, ACCCT=11, ACTCC=43	54	12,8	0.00001
23	MM4	chr9:21970916	C/T	C	SNV	CDKN2A	exonic	2	c.442G>A	p.Ala148Thr	missense	Benign	403	C=389, T=14	14	3,5	0.01866
23	MM4	chr3:178952037	C/T	C	SNV	PIK3CA	exonic	21	c.3092C>T	p.Thr1031Ile	missense	Likely pathogenic	915	C=885, T=30	30	3,3	0.00405
23	MM4	chr17:7579891	G/A	G	SNV	TP53	exonic	2	c.22C>T	p.Pro8Ser	missense		532	G=510, A=22	22	4,1	0.00067
61	MM1	chr7:140453136	ACT/TCT	ACT	SNV	BRAF	exonic	15	c.1799T>A	p.Val600Glu	missense	pathogenic	1920	ACT=1425, TCT=495	495	25,8	0.00001
61	MM1	chr17:7577539	GG/AG	GG	SNV	TP53	exonic	7	c.742C>T	p.Arg248Trp	missense	probable pathogenic	1999	GG=1955, AG=44	87	4,4	0.00634
61	MM2	chr19:17945696	C/T	C	SNV	JAK3	exonic	16	c.2164G>A	p.Val722Ile	missense		1173	C=1151, T=22	44	3,8	0.01988
61	MM3	chr7:140453136	ACT/TCT	ACT	SNV	BRAF	exonic	15	c.1799T>A	p.Val600Glu	missense	pathogenic	1900	ACT=1170, TCT=730	730	38,4	0.00001
61	MM3	chr2:212587210	T/G	T	SNV	ERBB4	exonic	7	c.791A>C	p.Gln264Pro	missense		1041	T=974, G=67	67	6,4	0.00176
61	MM3	chr4:153247358	T/C	T	SNV	FBXW7	exonic	10	c.1444A>G	p.Thr482Ala	missense		2000	T=1932, C=68	68	3,4	0.01708
61	MM3	chr4:55962501	T/C	T	SNV	KDR	exonic	19	c.2623A>G	p.Thr875Ala	missense		1126	T=1081, C=45	45	4,0	0.01349
61	MM3	chr7:116339673	G/A	G	SNV	MET	exonic	2	c.535G>A	p.Ala179Thr	missense		1998	G=1925, A=73	73	3,7	0.01687
61	MM3	chr7:128846393	T/A	T	SNV	SMO	exonic	6	c.1229T>A	p.Leu410Gln	missense		1917	T=1830, A=87	87	4,5	0.00298
61	MM4	chr7:140453136	ACT/TCT	ACT	SNV	BRAF	exonic	15	c.1799T>A	p.Val600Glu	missense	pathogenic	1323	ACT=851, TCT=472	472	35,7	0.00001
61	MM4	chr17:7579473	G/C	G	SNV	TP53	exonic	4	c.214C>G	p.Pro72Ala	missense	Benign, uncertain significance	1404	G=1338, C=66	76	5,4	0.00235
61	MM5	chr7:140453136	ACT/TCT	ACT	SNV	BRAF	exonic	15	c.1799T>A	p.Val600Glu	missense	pathogenic	1478	ACT=928, TCT=550	550	37,2	0.00001
61	MM6	chr7:140453136	ACT/TCT	ACT	SNV	BRAF	exonic	15	c.1799T>A	p.Val600Glu	missense	pathogenic	1736	ACT=1153, TCT=583	583	33,6	0.00001
61	MM7	chr7:140453136	ACT/TCT	ACT	SNV	BRAF	exonic	15	c.1799T>A	p.Val600Glu	missense	pathogenic	1312	ACT=716, TCT=596	596	45,4	0.00001
61	MM8	chr7:140453136	ACT/TCT	ACT	SNV	BRAF	exonic	15	c.1799T>A	p.Val600Glu	missense	pathogenic	1370	ACT=890, TCT=480	480	35,0	0.00001
61	MM8	chr17:7579401	A/G	A	SNV	TP53	exonic	4	c.286T>C	p.Ser96Pro	missense	not provided	1230	A=1100, G=130	130	10,6	0.00003
66	MM1	chr7:140453136	ACT/TCT	ACT	SNV	BRAF	exonic	15	c.1799T>A	p.Val600Glu	missense	pathogenic	1152	ACT=884, TCT=268	268	23,3	0.00001
66	MM1	chr4:55593464	A/C	A	SNV	KIT	exonic	10	c.1621A>C	p.Met541Leu	missense	Benign, not provided	1997	A=1015, C=982	982	49,2	0.00001
66	MM1	chr17:7579470	CGG/CGC	CGG	SNV	TP53	exonic	4	c.215C>G	p.Pro72Arg	missense	Benign, uncertain significance	279	CGG=141, CGC=138	138	49,5	0.00001
66	MM2	chr5:112175338	CAAAG/CAAAA	CAAAG	SNV	APC	exonic	16	c.4051G>A	p.Ala1351Thr	missense		272	CAAAG=258, CAAAA=14	14	5,1	0.00201
66	MM2	chr7:140453136	ACT/TCT	ACT	SNV	BRAF	exonic	15	c.1799T>A	p.Val600Glu	missense	pathogenic	262	ACT=230, TCT=32	32	12,2	0.00001
66	MM2	chr19:1220505	G/A	G	SNV	STK11	splicesite_3	4	splicesite_3				296	G=276, A=20	20	6,8	0.00172
66	MM2	chr17:7578395	GG/AG	GG	SNV	TP53	exonic	5	c.535C>T	p.His179Tyr	missense		306	GG=288, AG=18	18	5,9	0.00187
66	MM3	chr3:178927410	A/G	A	SNV	PIK3CA	exonic	7	c.1173A>G	p.Ile391Met	missense	unknown significance	595	A=250, G=345	345	58,0	0.00001
66	MM3	chr17:7579470	CGC/CGC	CGG	SNV	TP53	exonic	4	c.215C>G	p.Pro72Arg	missense	Benign, uncertain significance	456	CGC=21, CGC=435	435	95,4	0.00013
66	MM3	chr17:7577082	C/T	C	SNV	TP53	exonic	8	c.856G>A	p.Glu286Lys	missense		438	C=415,T=23	23	5,3	0.00968
66	MM4	chr7:140453136	ACT/TCT	ACT	SNV	BRAF	exonic	15	c.1799T>A	p.Val600Glu	missense	pathogenic	701	ACT=469, TCT=232	232	33,1	0.00001
66	MM4	chr3:178927410	A/G	A	SNV	PIK3CA	exonic	7	c.1173A>G	p.Ile391Met	missense	unknown significance	692	A=320, G=372	372	53,8	0.00001
66	MM5	chr7:140453136	ACT/TCT	ACT	SNV	BRAF	exonic	15	c.1799T>A	p.Val600Glu	missense	pathogenic	507	ACT=344, TCT=163	163	32,1	0.00001
66	MM5	chr4:55946182	C/T	C	SNV	KDR	exonic	30	c.3997G>A	p.Gly1333Arg	missense	not provided	356	C=341, T=15	15	4,2	0.01879
66	MM5	chr3:178927410	A/G	A	SNV	PIK3CA	exonic	7	c.1173A>G	p.Ile391Met	missense	unknown significance	401	A=169, G=232	232	57,9	0.00001
66	MM5	chr17:7579470	CGG/CGC	CGG	SNV	TP53	exonic	4	c.215C>G	p.Pro72Arg	missense	Benign, uncertain significance	339	CGG=27, CGC=312	312	92,0	0.00012
98	MM1	chr1:115258747	C/T	C	SNV	NRAS	exonic	2	c.35G>A	p.Gly12Asp	missense	Pathogenic	2000	C=1858, T=142	142	7,1	0.00001
98	MM1	chr3:178952078	G/T	G	SNV	PIK3CA	exonic	21	c.3133G>T	p.Asp1045Tyr	missense		521	G=498, T=23	23	4,4	0.00022
98	MM2	chr3:178927410	G/G	A	SNV	PIK3CA	exonic	7	c.1173A>G	p.Ile391Met	missense	Likely benign,not provided	2000	A=4, G=1996	1996	99,8	0.00001
98	MM2	chr3:178928119	CAAATAA/CAAATA	CAAATAA	INDEL	PIK3CA	exonic	8	c.1404delA	p.Lys468fs	frameshiftDeletion		1942	CAAATAA=1490, CAAATA=448, CAAAT=4	452	23,3	0.00001
98	MM2	chr17:7579472	G/C	G	SNV	TP53	exonic	4	c.215C>G	p.Pro72Arg	missense	Benign,Uncertain significance	1974	G=958, C=1016	1016	51,5	0.00001
98	MM3	chr1:115258747	C/T	C	SNV	NRAS	exonic	2	c.35G>A	p.Gly12Asp	missense	Pathogenic	1118	C=982, T=136	136	12,2	0.00001
98	MM3	chr3:178947832	G/A	G	SNV	PIK3CA	exonic	19	c.2707G>A	p.Gly903Arg	missense		900	G=842, A=58	58	6,4	0.00001
98	MM3	chr17:7577106	G/A	G	SNV	TP53	exonic	8	c.832C>T	p.Pro278Ser	missense	Likely pathogenic,Uncertain significance	386	G=370, A=16	16	4,1	0.00272
98	MM4	chr4:55972974	T/A	T	SNV	KDR	exonic	11	c.1416A>T	p.Gln472His	missense	Likely benign,not provided	1304	T=677, A=627	627	48,1	0.00001
98	MM4	chr3:178952090	G/A	G	SNV	PIK3CA	exonic	21	c.3145G>A	p.Gly1049Ser	missense	Likely pathogenic	607	G=580, A=27	27	4,4	0.00006
98	MM5	chr4:55972974	T/A	T	SNV	KDR	exonic	11	c.1416A>T	p.Gln472His	missense	Likely benign,not provided	319	T=264, A=55	55	17,2	0.00001
98	MM5	chr3:178927410	G/G	A	SNV	PIK3CA	exonic	7	c.1173A>G	p.Ile391Met	missense	Likely benign,not provided	890	A=18, G=872	872	98,0	0.00001
98	MM5	chr17:7579469	ACGGG/ACGCG	ACGGG	SNV	TP53	exonic	4	c.215C>G	p.Pro72Arg	missense	Benign,Uncertain significance	379	ACGGG=107, ACGAG=7, ACGCA=5, ACGCG=218	230	60,7	0.00001
146	MM1	chr7:140453136	ACT/TCT	ACT	SNV	BRAF	exonic	15	c.1799T>A	p.Val600Glu	missense	pathogenic	1152	ACT=884, TCT=268	268	23,3	0.00001
146	MM1	chr4:55593464	A/C	A	SNV	KIT	exonic	10	c.1621A>C	p.Met541Leu	missense	Benign, not provided	1997	A=1015, C=982	982	49,2	0.00001
146	MM1	chr17:7578263	GG/AA	GG	MNV	TP53	exonic	6	c.585_586delCCinsTT	p.Arg196Ter	nonsense	probable pathogenic	884	GG=478, AA=406	406	45,9	0.00001
146	MM1	chr17:7579470	CGG/CGC	CGG	SNV	TP53	exonic	4	c.215C>G	p.Pro72Arg	missense	Benign, uncertain significance	279	CGG=141, CGC=138	138	49,5	0.00001

Case #	*Sample*	*Locus*	*Genotype*	*Ref*	*Type*	*Gene*	*Location*	*Exon*	*Coding*	*Protein*	*Variant effect*	*Clinvar/COSMIC*	*Coverage*	*Allele coverage*	*Mutated allele*	*Mutated allele %*	*pvalue*
**146**	**MM2**	**chr7:140453135**	**CA/TT**	**CA**	**MNV**	**BRAF**	**exonic**	**15**	**c.1799T>A**	**p.Val600Glu**	**missense**	**pathogenic**	**700**	**CA=272, TT=428**	**428**	**61,1**	**0.00001**
146	MM2	chr7:140453133	T/A	T	SNV	BRAF	exonic	15	c.1802A>T	p.Lys601Ile	missense	other	705	T=277, A=428	428	60,7	0.00001
**146**	**MM3**	**chr7:140453136**	**A/T**	**A**	**SNV**	**BRAF**	**exonic**	**15**	**c.1799T>A**	**p.Val600Glu**	**missense**	**pathogenic**	**782**	**A=486, T=296**	**296**	**37,9**	**0.00001**
146	MM3	chr3:178916932	A/C	A	SNV	PIK3CA	exonic	2	c.319A>C	p.Asn107His	missense	not provided	500	A=477, C=23	23	4,6	0.00212
146	MM3	chr17:7579472	G/C	G	SNV	TP53	exonic	4	c.215C>G	p.Pro72Arg	missense	Benign, uncertain significance	768	G=148, C=418	418	54,4	0.00001
**146**	**MM4**	**chr7:140453136**	**A/T**	**A**	**SNV**	**BRAF**	**exonic**	**15**	**c.1799T>A**	**p.Val600Glu**	**missense**	**pathogenic**	**1629**	**A=1101, T=528**	**528**	**32,4**	**0.00001**
146	MM4	chr17:7579472	G/C	G	SNV	TP53	exonic	4	c.215C>G	p.Pro72Arg	missense	Benign, uncertain significance	1038	G=477, C=502	502	48,4	0.00001
**146**	**MM5**	**chr7:140453136**	**A/T**	**A**	**SNV**	**BRAF**	**exonic**	**15**	**c.1799T>A**	**p.Val600Glu**	**missense**	**pathogenic**	**975**	**A=805, T=170**	**170**	**17,4**	**0.00001**
146	MM5	chr4:55972974	T/A	T	SNV	KDR	exonic	11	c.1416A>T	p.Gln472His	missense	Benign, uncertain significance	934	T=495, A=439	439	47,0	0.00001
146	MM5	chr4:55593464	C/C	A	SNV	KIT	exonic	10	c.1621A>C	p.Met541Leu	missense	Benign, not provided	1663	A=17, C=1646	1646	99,0	0.00001
**146**	**MM6**	**chr7:140453136**	**A/T**	**A**	**SNV**	**BRAF**	**exonic**	**15**	**c.1799T>A**	**p.Val600Glu**	**missense**	**pathogenic**	**1298**	**A=649, T=649**	**649**	**50,0**	**0.00001**
146	MM6	chr4:55972974	T/A	T	SNV	KDR	exonic	11	c.1416A>T	p.Gln472His	missense	Benign, uncertain significance	903	T=348, A=555	555	61,5	0.00001
146	MM6	chr4:55593464	C/C	A	SNV	KIT	exonic	10	c.1621A>C	p.Met541Leu	missense	Benign, not provided	1148	A=1, C=1147	1147	99,9	0.00001
